# Manipulating the ordered oxygen complexes to achieve high strength and ductility in medium-entropy alloys

**DOI:** 10.1038/s41467-023-36319-0

**Published:** 2023-02-13

**Authors:** Meiyuan Jiao, Zhifeng Lei, Yuan Wu, Jinlong Du, Xiao-Ye Zhou, Wenyue Li, Xiaoyuan Yuan, Xiaochun Liu, Xiangyu Zhu, Shudao Wang, Huihui Zhu, Peipei Cao, Xiongjun Liu, Xiaobin Zhang, Hui Wang, Suihe Jiang, Zhaoping Lu

**Affiliations:** 1 Beijing Advanced Innovation Center for Materials Genome Engineering, State Key Laboratory for Advanced Metals and Materials, University of Science and Technology Beijing, 100083 Beijing, China; 2 College of Materials Science and Engineering, Hunan University, 410082 Changsha, China; 3 Electron Microscopy Laboratory, School of Physics, Peking University, 100871 Beijing, China; 4 Guangdong Province Key Laboratory of Durability for Marine Civil Engineering, School of Civil Engineering, Shenzhen University, 518060 Shenzhen, China; 5 Institute of Metals, College of Materials Science and Engineering, Changsha University of Science & Technology, 410114 Changsha, China; 6 Department of Materials Science and Engineering, The University of Texas at Dallas, Richardson, TX 75080 USA

**Keywords:** Metals and alloys, Mechanical properties

## Abstract

Oxygen solute strengthening is an effective strategy to harden alloys, yet, it often deteriorates the ductility. Ordered oxygen complexes (OOCs), a state between random interstitials and oxides, can simultaneously enhance strength and ductility in high-entropy alloys. However, whether this particular strengthening mechanism holds in other alloys and how these OOCs are tailored remain unclear. Herein, we demonstrate that OOCs can be obtained in bcc (body-centered-cubic) Ti-Zr-Nb medium-entropy alloys via adjusting the content of Nb and oxygen. Decreasing the phase stability enhances the degree of (Ti, Zr)-rich chemical short-range orderings, and then favors formation of OOCs after doping oxygen. Moreover, the number density of OOCs increases with oxygen contents in a given alloy, but adding excessive oxygen (>3.0 at.%) causes grain boundary segregation. Consequently, the tensile yield strength is enhanced by ~75% and ductility is substantially improved by ~164% with addition of 3.0 at.% O in the Ti-30Zr-14Nb MEA.

## Abstract (web summary)

Ordered oxygen complexes (OOCs) endow a unique interstitial strengthening mechanism for simultaneously enhancing strength and ductility in HEAs. Here, the authors demonstrate whether such mechanism can be extended to other alloy systems and how the formation of OOCs is tailored.

## Introduction

Interstitial solutes, such as oxygen, nitrogen, carbon, and boron, are very effective strengtheners in metals and alloys, i.e., interstitial strengthening^1–6^. In body-centered cubic (bcc) alloys, the interstitial atoms fill the octahedral or tetrahedral interstitial vacancies^7^, and both kinds of occupations enforce a local deformation with tetragonal symmetry and produce tetragonal distortions of the lattice. Due to the relatively large shear strain associated with the tetragonal defects, the interaction between screw dislocations and tetragonal distortions is substantial. Therefore, the strengthening potency of interstitials is generally much larger than that of substitutional solutes which typically produce a spherically symmetric stress field around the solute^8^. Conventional wisdom has assumed that interstitials occupy interstices randomly, and their strengthening in metals and alloys often comes with a significant sacrifice of ductility^9–12^. Such behavior can be related to the significant distortions of the host matrix at these microstructural sites, promoting local decohesion and inducing localized plastic fracture^13,14^.

Our recent finding revealed that in the TiZrHfNb high-entropy alloy (HEA), oxygen can assume the constitutive form of ordered oxygen complexes (OOCs), a state between regular random interstitials and oxides. The OOCs not only offer interstitial strengthening of oxygen atoms which significantly increases the strength, but also work as dislocation pinning centers, change the dislocation shear mode from planar slip to wavy slip and significantly enhanced the ductility. Strikingly, OOCs avoid the undesirable embrittlement effects of interstitial atoms and offer an approach to concurrently increasing strength and ductility^15,16^. Nevertheless, how to design strong and ductile metallic materials based on the formation of OOCs still remains unclear. Therefore, it is urgent to elucidate the mechanisms for manipulating the OOCs so that such strengthening mechanism can be extended to not only HEAs, but also MEAs and traditional alloys.

In this study, we selectet a series of Ti–30Zr–*x*Nb (*x* = 10, 14, 18, 22, 26, and 30 at.%) MEAs as model alloys, and by substituting Ti with a different amount of oxygen, we systematically investigate the formation behavior of OOCs and its effects on mechanical properties. We find that the existence of sufficient (Ti, Zr)-enriched chemical short-range orderings (CSROs) is a prerequisite for forming OOCs, and that the oxygen concentration has to be carefully optimized to achieve the maximal ductilization efficiency with no occurrence of segregation. As a result, we develop the Ti-30Zr-14Nb-3O MEA which exhibits the optimal strength-ductility synergy with a tensile yield strength of ~1075 MPa and elongation of ~25%. The tensile yield strength is enhanced by ~75% while the ductility is substantially improved by ~164%, as compared to the base Ti-30Zr-14Nb MEA with no addition of oxygen. The work here provides a practical guide for developing strong yet ductile metallic alloys which can dissolve a certain amount of oxygen.

## Results

### Tensile properties

**Fig. 1:**
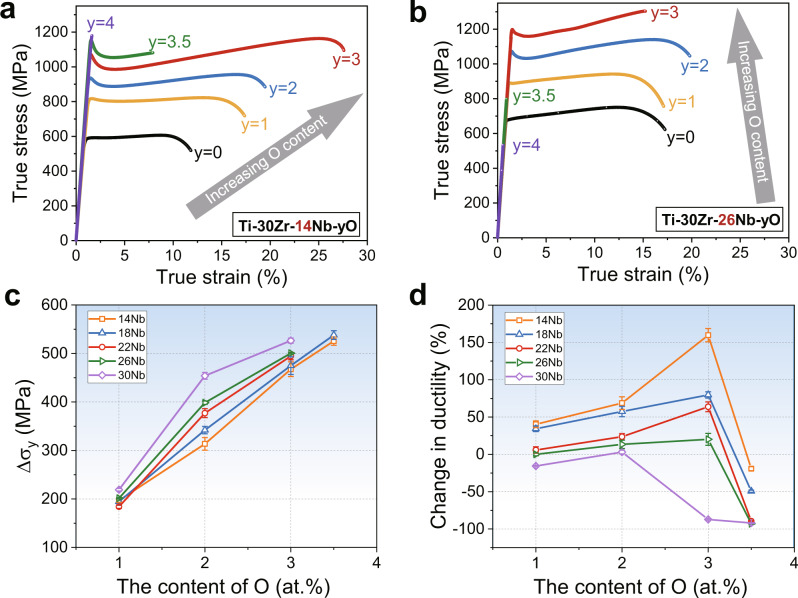
Mechanical properties. Room-temperature tensile stress-strain curves of the as-cast (**a**) Ti–30Zr–14Nb-*y*O and (**b**) Ti–30Zr–26Nb–*y*O (*y* = 0, 1, 2, 3, 3.5, and 4 at.%) MEAs. **c** The yield strength increments (*∆σ*_*y*_) of Ti–30Zr–*x*Nb (*x* = 14, 18, 22, 26, and 30 at.%) MEAs after alloying different levels of oxygen. **d** The change in ductility varies with oxygen contents in Ti-30Zr-xNb MEAs.

Tensile properties of Ti–30Zr–*x*Nb–*y*O (*x* = 10, 14, 18, 22, 26, and 30 at.%, and y = 0, 1, 2, 3, 3.5, and 4 at.%) MEAs were tested at room temperature with a strain rate of 10^−3^ s^−1^. Figure 1a, b exhibits typical true tensile stress-strain curves of Ti–30Zr–14Nb–*y*O and Ti–30Zr–26Nb–*y*O MEAs while Fig. 1c, d illustrates dependence of the yield strength and ductility as a function of Nb and O contents for all the alloys investigated. Interestingly, unlike the conventional interstitial strengthening behavior, the addition of 1–3 at.% oxygen in the Ti–30Zr–14Nb MEA not only increases the yield strength but also significantly improves the ductility (see Fig. 1a). Particularly, the elongation is nearly enhanced by ~164%, from ~9.5% for the Ti–30Zr–14Nb base MEA to ~25.1% for the Ti–30Zr–14Nb–3O variant. When the oxygen content exceeds 3.5 at.%, the ductility declines until a brittle fracture occurs. Ti–30Zr–18Nb–*y*O and Ti–30Zr–22Nb–*y*O MEAs show a similar trend (see Supplementary Fig. 1b, c). However, for the Ti–30Zr–26Nb and Ti–30Zr–30Nb MEAs, the ductility increases slightly with the increase of oxygen content, as shown in Fig. 1b and Supplementary Fig. 1d, and excessive addition of oxygen causes obvious embrittlement. Notably, a yield-drop phenomenon was recorded in the tensile stress-strain curves of these MEAs after doping oxygen, which is similar to that observed for the low-carbon steels and the oxygen-doped TiZrHfNb HEAs^15,17^. It should be noted that the ductility dramatically deteriorates with oxygen concentration in the Ti–30Zr–10Nb MEA (see Supplementary Fig. 1a). The loss of ductility might originate from suppression of the martensite phase transformation or/and twin formation, as reported previously in some dual-phase titanium alloys^9,18,19^.

In the alloys with a given amount of Nb from 14 to 30 at.%, the yield strength increases with the oxygen content (Fig. 1c), demonstrating strong interstitial strengthening. For the ductility wise, when the Nb content is below 26 at.%, it increases with the oxygen concentration (i.e., the ductility change ratio of the doped MEA to the un-doped counterpart is positive) and reaches its maximum in the sample with 3.0 at.% oxygen (Fig. 1d). It is interesting to note that the strongest ductilization effect of oxygen was realized in the alloy with 14 at.% Nb, i.e., with addition of 3 at.% oxygen, the ductility is increased by 164 %.

### Tuning mechanisms of OOCs

**Fig. 2:**
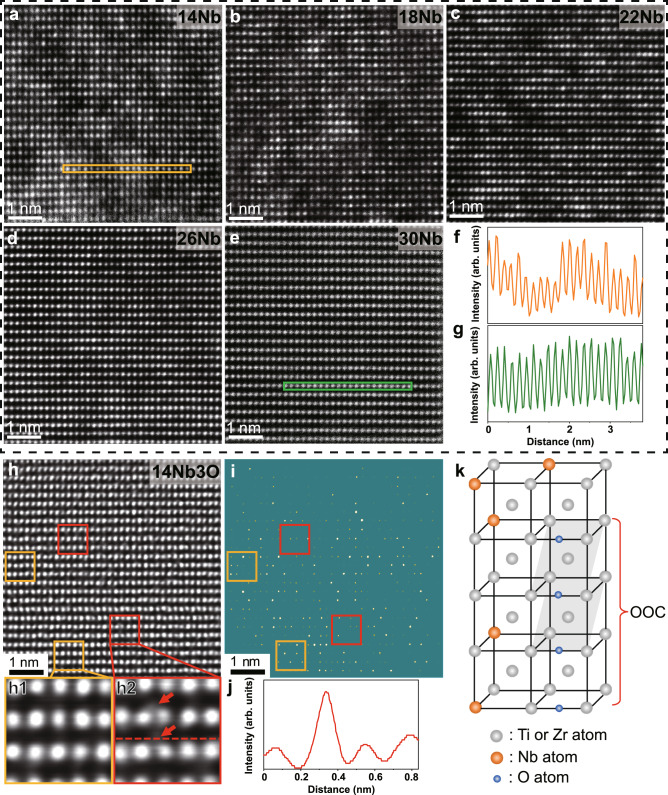
Chemical short-range orderings and ordered oxygen complexes (OOCs). **a**–**e** STEM-HAADF images for the [011]_bcc_ crystal axis in the Ti–30Zr–*x*Nb (*x* = 14, 18, 22, 26, and 30 at.%) MEAs. The degree of chemical short-range orderings decreases with increasing Nb content. **f**, **g** Intensity line profiles corresponding to the orange and green squared regions in (**a**) and (**e**), respectively, indicating a reduced chemical inhomogeneity in Ti–30Zr–30Nb MEA than that in Ti–30Zr–14Nb MEA. **h**, **i** iDPC images for the [011]_bcc_ crystal axis with different adjusted contrast to reveal the existence of chemical short-range orderings in the Ti–30Zr–14Nb–3O MEA. Red squares represent the Ti/Zr-rich regions and orange squares indicate the Nb-rich regions. **h1**, **h2** Enlargements of the orange and red squares in (**h**), revealing the formed OOCs. **j** Intensity line profile of the red dashed line in (**h2**), which is obtained by the standard tool of Digital Microscopy software. **k** Schematic diagram of the OOC. Gray, orange and blue bubbles represent Ti/Zr, Nb, and O atoms, respectively.

To reveal the underlying mechanism of the different interstitial strengthening behaviors of oxygen in these materials, we characterized microstructures of all the MEAs in detail down to the atomic scale. The Ti–30Zr–10Nb MEA shows a dual-phase structure of bcc+α“, whilst a single bcc phase exists in Ti–30Zr–xNb with a larger amount of Nb (i.e., *x* = 14, 18, 22, 26, and 30 at.%) (Supplementary Fig. 2). Figure 2a–e illustrates the aberration-corrected scanning transmission electron microscope high-angle annular dark-field (STEM-HAADF) images of Ti–30Zr–xNb (*x* = 14, 18, 22, 26, and 30 at.%) MEAs taken along the [110] zone axis. The *Z*-contrast image (where *Z* is the atomic number) is immensely sensitive to local changes in the atomic numbers of the constituent elements^20^. Namely, light atoms exhibit dark contrast while heavy atoms are imaged bright. The *Z*-contrast distribution of the STEM-HAADF image in Fig. 2a clearly demonstrates the existence of two types of local regions in the Ti–30Zr–14Nb MEA; one is rich in light atoms, i.e., (Ti, Zr)-rich, and the other is enriched with heavy atoms (i.e., Nb-rich). This observation manifests the formation of CSROs, similar to that reported in HEAs previously^15,21^. However, with the increase of Nb, the atomic-scale inhomogeneity gradually become imperceptible (see Fig. 2b–e). Figure 2f, g shows intensity profiles corresponding to the orange and green rectangular regions in Fig. 2a, e, respectively, further confirming that the intensity fluctuations in Ti–30Zr–14Nb MEA are more pronounced than those in Ti–30Zr–30Nb MEA. The above results suggest that the degree of CSROs decreases with the increase of Nb in the current alloy system due to the enhancement in the phase stability with the addition of more bcc stabilizers. The geometric phase analysis of the Ti–30Zr–14Nb and Ti–30Zr–30Nb MEAs also verifies this point (Supplementary Fig. 3a–f). Moreover, the Warren–Cowley parameters calculated by *ab* initio molecular dynamics simulations confirm that the formation of the Ti–Zr and Nb–Nb pairs is more favorable in Ti–30Zr–14Nb than in Ti–30Zr–30Nb (Supplementary Fig. 3g), which is consistent with the experimental observations.

To identify the occupancy state of oxygen atoms, the integrated differential phase contrast STEM (iDPC-STEM) images were taken, and Fig. 2h shows the typical result of Ti–30Zr–14Nb–3O MEA. The iDPC-STEM characterization is a low-dose technique with a high signal-to-noise ratio, and more strikingly, this technique can well preserve the intensity ratio of the light to the heavy element columns^22,23^. In other words, this method can simultaneously image light and heavy atoms, especially showing decent visibility of interstitial elements on a black background^24–26^. To make each atomic column more clearly presented, the contrast of the image is tuned. As shown in Fig. 2i, the existence of the regions enriched in heavy atoms (that is, Nb-rich) and those in light atoms (i.e., (Ti, Zr)-rich) can be easily identified, as highlighted by orange and red squares, respectively. The enlarged views of the relevant regions are shown in Fig. 2h1, h2, respectively. In contrast to the Nb-enriched region (i.e., the orange square in Fig. 2h), the (Ti, Zr)-enriched region (i.e., the red square in Fig. 2h) has some small light dots (marked by the red arrows) inserted in the lattice. This observation reveals that oxygen atoms occupy the octahedral interstitial sites in the bcc lattice. Figure 2j shows the intensity line profile corresponding to the red dashed line in Fig. 2h2. One atomic column exhibits a sharp increase in intensity, which further verifies the interstitial occupation of oxygen atoms. Statistical analysis of the iDPC-STEM images (Fig. 2h, i) indicates that oxygen prefers to occupy interstitial positions adjacent to light-atom-rich (i.e., Ti and/or Zr) lattice sites and form the so-called OOCs^15^, as schematically shown in Fig. 2k. Furthermore, atom probe tomography measurements of the Ti–30Zr–14Nb–3O MEA also affirm the presence of OOCs (see Supplementary Fig. 4). The slight enrichment of Ti and Zr atoms and the corresponding depletion of Nb atoms were observed within the OOCs, which is consistent with the iDPC-STEM result.

**Fig. 3:**
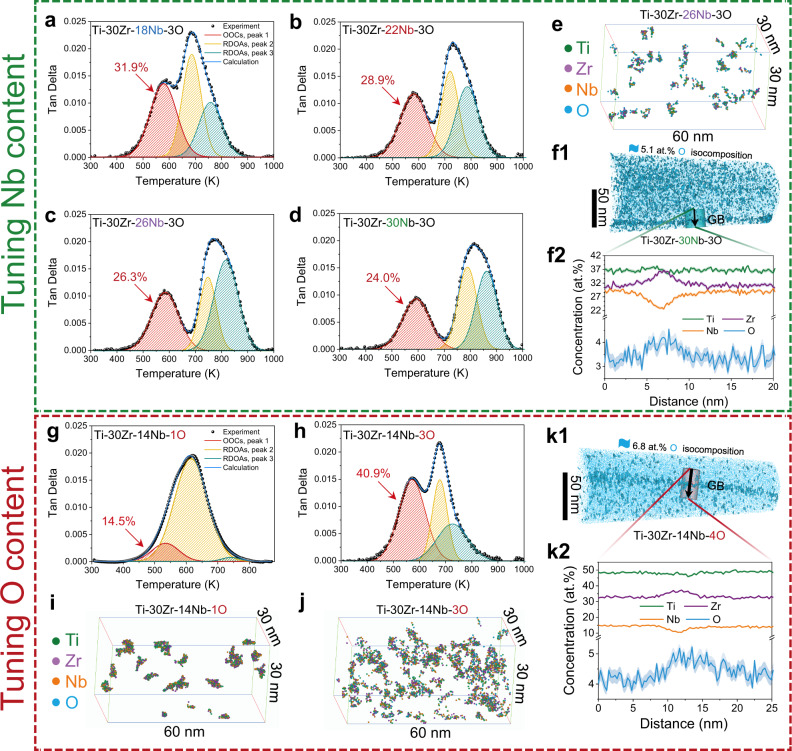
Regulation of OOCs by tuning Nb and O content. Temperature dependence of internal friction (at 1.0 Hz) and the fitting results in the (**a**–**d**) Ti–30Zr–*x*Nb-3O (*x* = 18, 22, 26 and 30 at.%) and (**g**, **h**) Ti–30Zr–14Nb–*y*O (*y* = 1 and 3 at.%) MEAs. The solid black circles and the blue curves are the experimental results and the sum of the cumulative fitting peaks, respectively. The yellow and green curves correspond to the relaxation processes of the randomly distributed oxygen atoms (RDOAs). The red curves indicate the reorientation of OOCs. The calculated percentages of the oxygen atoms involved in the formation of OOCs are indicated by red arrows. Fewer oxygen atoms participate in the formation of OOCs with increasing Nb content. More oxygen atoms in Ti–30Zr–14Nb–3O participate in the formation of OOCs than that in Ti–30Zr–14Nb–1O MEA. **e**, **i**, **j** The 3D reconstruction of OOCs obtained via cluster analysis in Ti–30Zr–26Nb–3O, Ti–30Zr–14Nb–1O, and Ti–30Zr–14Nb–3O MEAs. Green, purple, orange, and blue colors correspond to Ti, Zr, Nb, and O atoms, respectively. **f1**, **k1** 3D APT tip reconstruction of the Ti–30Zr–30Nb–3O and Ti–30Zr–14Nb–4O MEAs, respectively. The thresholds for the isocomposition surface are 5.1 at.% and 6.8 at.%, respectively. Oxygen segregates at grain boundaries (GB). Other elements were hidden for a simplified illustration. **f2**, **k2** The corresponding 1D concentration profiles across the grain boundaries (black arrows in **f1** and **k1)**.

**Fig. 4:**
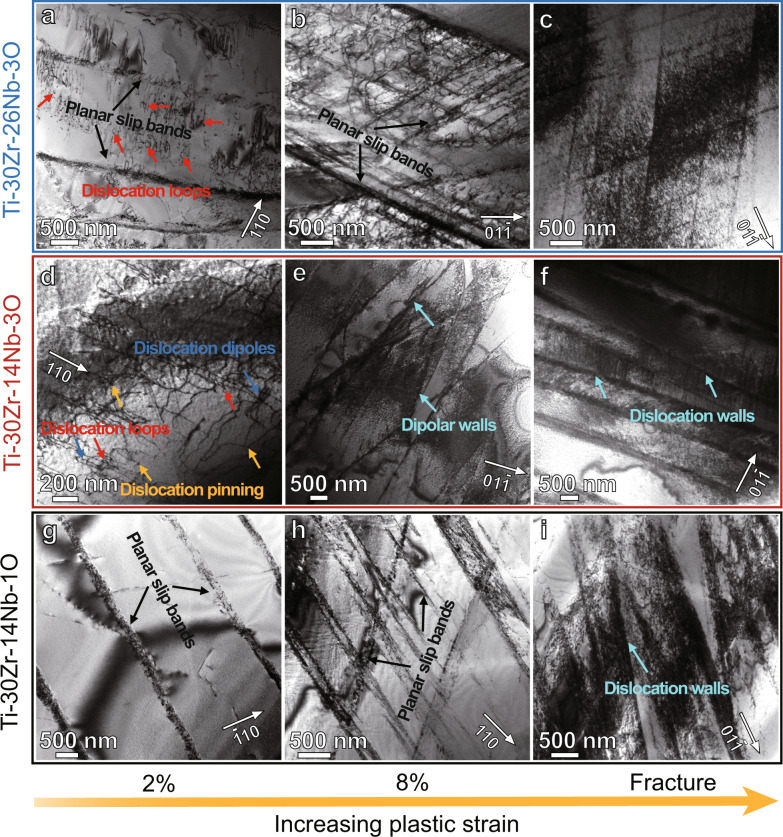
Dislocation morphologies of pre-strained and fractured samples of typical MEAs. **a**–**c** Ti-30Zr-26Nb-3O, (**d**–**f**) Ti-30Zr-14Nb-3O and (**g**–**i**) Ti–30Zr–14Nb–1O MEAs. The white arrow with the **g** vector marks the two-beam diffraction condition utilized. The black and red arrows indicate the planar slip bands and dislocation loops, respectively. The yellow and blue arrows denote the dislocation pinning and dislocation dipoles, respectively. The dipolar walls and the dislocation walls are highlighted by cyan arrows. For the Ti–30Zr–26Nb–3O and Ti–30Zr–14Nb–1O MEAs, planar slip prevails at the initial stage and wavy slip occurs at the late stage. While for the Ti–30Zr–14Nb–3O MEA, wave slip dominates the entire deformation process.

Internal-friction measurements were also conducted to further understand the effect of Nb and oxygen on the formation of OOCs, and representative spectra for the Ti–30Zr–14Nb–1O and Ti–30Zr–*x*Nb-3O (*x* = 14, 18, 22, 26, and 30 at.%) MEAs are shown in Supplementary Fig. 5. By decomposing the relaxation structure following the method proposed previously, we estimated the proportion of the oxygen atoms which involved in the formation of OOCs in oxygen-doped alloys^16^. A low-temperature peak (red curves) and two high-temperature peaks (yellow and green curves) can be observed in all the oxygen-containing MEAs (see Fig. 3a–d, 3g, h). The formation of OOCs spawns the low-temperature peak, whilst the randomly distributed oxygen atoms produce the two high-temperature peaks^16^. For the alloys with the same level of oxygen, i.e., Ti–30Zr–xNb–3O (*x* = 14, 18, 22, 26, and 30 at.%) MEAs, the calculated percentage of the oxygen atoms involved in the formation of OOCs approximately is 40.9, 31.9, 28.9, 26.3, and 24.0%, respectively. The ratio decreases as the Nb content rises, suggesting that fewer oxygen atoms are engaged in the formation of OOCs. Additionally, we obtained three-dimensional APT reconstruction of clusters via cluster analysis in the box with a volume of 30 × 30 × 60 nm^3^. The cluster analysis parameters, *d*_max_ and N_min_, for Ti–30Zr–14Nb–3O and Ti–30Zr–26Nb–3O have been listed in Supplementary Table 1. The number density of OOCs decreases from 8.7 × 10^24^ m^−3^ for the former (Fig. 3j) to 6.7 × 10^23^ m^−3^ for the latter (Fig. 3e). The iDPC-STEM images of both MEAs show that oxygen atomic columns are more frequently observed in Ti-30Zr-14Nb-3O (see Supplementary Fig. 6a, b) than that in Ti–30Zr–26Nb–3O (see Supplementary Fig. 6c, d), indicating that the formed amount of OOCs is reduced in Ti–30Zr–26Nb–3O. Moreover, in the high Nb-containing specimens, e.g., Ti–30Zr–30Nb–3O, the 5.1 at.%-O iso-concentration surface and the corresponding 1D concentration map clearly visualize a higher oxygen concentration specifically at the grain boundary relative to the matrix, revealing that the oxygen atoms were segregated at the grain boundary (Fig. 4f1, f2) due to excessive addition of oxygen. Based on the above results, it is clear that the number density of OOCs decreases with the increase of Nb, and so does the solubility of oxygen in the matrix.

As described above, the oxygen content also affects the formation of OOCs. Figure 3g, h shows internal-friction spectra (at 1.0 Hz) of the alloys with 14 at.% Nb but a different amount of 1 and 3 at.% O, i.e., Ti–30Zr–14Nb–1O and Ti–30Zr–14Nb–3O. After the peak decomposition analysis, it is known that the ratio of oxygen atoms participating in forming OOCs increases with the oxygen concentration, i.e., from ~14.5% to ~40.9% as the oxygen content is raised from 1 to 3 at.%. As such, the number density of the clusters increases substantially from 1.04 × 10^24^ m^−3^ for Ti–30Zr–14Nb–1O MEA (Fig. 3i) to 8.7 × 10^24^ m^−3^ for Ti–30Zr–14Nb–3O MEA (Fig. 3j). Moreover, in the low oxygen-doped Ti–30Zr–14Nb–1O MEA (see Supplementary Fig. 6e, f), fewer oxygen atomic columns were observed in the interstitial sites within light-atom-rich regions, indicating a reduction in the amount of OOCs with the decrease of doped O content. With the further increase of oxygen, i.e., the Ti–30Zr–14Nb–4O MEA, the 6.8 at.%-O iso-composition surfaces and the corresponding 1D concentration map also reveal the occurrence of grain boundary segregation of oxygen (Fig. 3k1, k2).

### Contribution of OOCs to deformation behavior

We confirmed that no phase transformation or twin formation occurred in Ti–30Zr–14Nb–1O, Ti–30Zr–14Nb–3O and Ti–30Zr–26Nb–3O MEAs (Supplementary Fig. 7a). The corresponding XRD patterns of the fractured samples further demonstrate no phase transformation during deformation (Supplementary Fig. 7b), indicating that deformation of these three MEAs proceeds mainly via dislocation glide. To clarify how the number density of OOCs, i.e, the oxygen atomic occupation, affects the plastic deformation behavior and ductility of the current MEAs, we conducted a detailed analysis of the dislocation features formed during loading. Figure 4 illustrates the dislocation substructures of the Ti–30Zr–26Nb–3O, Ti–30Zr–14Nb–3O and Ti–30Zr–14Nb–1O specimens strained to different stages. Two-beam bright-field images were taken under different **g** vectors to determine the dislocation types, where **g** is the vector of the reflecting lattice planes^27^. For the Ti–30Zr–26Nb–3O MEA, planar slip bands become predominated after 2% strain, but numerous jagged dislocations and tangles were generated between the bands (Fig. 4a), indicating the occurrence of cross-slip events inside the planar slip bands. After 8% straining, more planar slip bands and dislocation tangles appeared (Fig. 4b), whilst after the fracture, a high density of forest dislocations emerged throughout the matrix (Fig. 4c), although the planar slip bands were still present. Apparently, during the deformation of Ti–30Zr–26Nb–3O MEA, planar slip was dominant at the initial stage and wavy slip prevailed at the late stage. By contrast, for the Ti–30Zr–14Nb–3O MEA, dislocation loops (red arrows) and dipoles (blue arrows) were formed after 2% strain (Fig. 4d), manifesting strong interaction between the OOCs and dislocations. The pinning effects assisted by OOCs also exist in the matrix (see yellow arrows). With the increase of strain to 8%, dipolar walls caused by cross-slip of screw dislocations appeared (Fig. 4e). After rupture, deformation delocalization continues, leading to a more homogenous distribution of dislocations. Some dislocation walls interweaving with each other are also observed (Fig. 4f), and the plastic deformation of the Ti–30Zr–14Nb–3O MEA is clearly characterized by frequent dislocation cross-slip and dominated by wave slip. For the Ti–30Zr–14Nb–1O MEA, we can see planar slip bands in the sample strained to 2% (Fig. 4g) and dense dislocation networks of planar dislocation arrays in the specimen strained to 8% (Fig. 4h). Several dislocation walls caused by dislocation cross-slips are also observed in the fractured sample of this MEA (Fig. 4i), showing a typical cell-forming deformation structure^28^. These results suggest that the deformation of Ti–30Zr–14Nb–1O MEA is controlled by planar slip at the initial stage and wavy slip at the subsequent stage. It is worth mentioning here that the plastic deformation mode of Ti–30Zr–14Nb MEA is characterized by planar slip (Supplementary Fig. 8). Based on the above observations, we clarify that the deformation mode of the current MEAs changes from planar slip to wavy slip with the increase in the number density of OOCs.

## Discussion

As described above, we can effectively manipulate the formation of OOCs to simultaneously obtain high strength and large ductility via tuning Nb and O contents in the Ti–Zr–Nb–O MEAs. To clarify the intrinsic formation mechanism of OOCs in the current MEAs, we investigated compositional effects from the standpoint of electronic structure by density functional theory (DFT) calculations (see Supplementary Discussion and Supplementary Fig. 9). We found that Nb atoms are prone to bond with other Nb atoms, rather than Ti and Zr atoms (Supplementary Fig. 9a, b). The total number of Ti–Ti, Ti–Zr, and Zr–Zr bonds in Ti-30Zr-30Nb is much less than that in Ti–30Zr–14Nb, which is consistent with the experimental observations that the degree of (Ti, Zr)-CSROs decreased with the increase of Nb. After doping oxygen, O solution energy decreases as more Ti and Zr atoms occupy the nearest neighbors but increases significantly when the O atom is surrounded by Nb atoms (Supplementary Fig. 9c). Therefore, O atoms are more likely to occupy octahedral interstitial sites in the (Ti, Zr)-CSROs, resulting in the formation of OOCs. The bonding properties conducted by the Bader charge analysis^29^ also verify this point (see Supplementary Discussion and Supplementary Fig. 10, Table 2). Moreover, the O solution energies in Ti–30Zr–30Nb are higher than those in Ti–30Zr–14Nb (Supplementary Fig. 9d). In other words, a lower Nb composition in Ti–Zr–Nb MEAs (e.g., Ti–30Zr–14Nb) renders more solution sites for O than that in high-Nb containing MEA (e.g., Ti–30Zr–30Nb), resulting in a higher number density of OOCs when doping the same amount of oxygen.

Since these alloys consist of a single bcc phase (Supplementary Fig. 11), the main factors affecting their yield strength would be grain boundary strengthening and solid solution strengthening. To evaluate grain boundary strengthening, we employed the Hall-Petch equation^30,31^

1 $${\sigma }_{y}={\sigma }_{0}+K{d}^{-1/2},$$

where *σ*_*y*_, *σ*_0_, *d*, and *K* are the yield stress, frictional stress, grain size and material constant, respectively. The material constant *K* of the Ti–30Zr–14Nb and Ti–30Zr–26Nb MEAs were estimated to be 238 and 256 MPa µm^1/2^, respectively^32^. According to the grain size variation of the oxygen-doped MEAs (Supplementary Fig. 11), the calculated theoretical increments of yield strength are quite small (Supplementary Table 3). Thus, the changes in the yield strength of the current MEAs are not originated from grain boundary strengthening.

It is to be noticed that the calculated lattice parameters of both Ti–30Zr–14Nb–*y*O and Ti–30Zr–26Nb–*y*O (*y* = 0, 1, 2, 3, 3.5, 4 at.%) MEAs increase monotonically with the increase of O, implying that O atoms were solutionized in the matrix and increased the lattice distortion (Supplementary Fig. 11e, f). Here, due to the low contents of O, we then consider the oxygen-doped MEAs as diluted solutions. Therefore, the classical Fleischer model which works for dilute solutions^33^ was adopted to evaluate the effects of solid-solution strengthening in the current MEAs. As shown in Supplementary Fig. 12, the increment in the yield strength (Δσ_s_) of the oxygen-doped Ti–30Zr–*x*Nb (*x* = 14 and 26 at.%) MEAs follows a linear relationship with **c**^1/2^ (**c** is the content of O), confirming that this model can well describe the strengthening effect of oxygen addition.

It is well known that interstitial atoms usually occupy tetrahedral or octahedral interstitial sites in the bcc lattice, leading to a local deformation with tetragonal symmetry and producing tetragonal distortions of the lattice. The interaction between tetragonal distortions and screw dislocations is substantial due to the relatively large shear strain associated with tetragonal defects^34^. Fleischer regarded solid-solution hardening by such tetragonal distortion fields as ‘rapid hardening’ and estimated the yield strength increment to be:

2 $$\Delta \tau=\,\frac{G\Delta\varepsilon{c}^{1/2}}{3},$$

where $G$ is the shear modulus, Δ*ε* is the difference between the longitudinal and transverse strain of the tetragonal distortion source when interstitial atoms occupy the interstitial sites in alloys, and $c$ is the atomic concentration of interstitial atoms creating such defects. Herein, we use the change of hardness Δ*H*_*v*_ to quantify the effect of solid-solution strengthening^35^, rendering the above equation as

3 $$\Delta {H}_{v}={3}^{3/2}\frac{G\Delta\varepsilon{c}^{1/2}}{3}={3}^{1/2}G\Delta\varepsilon{c}^{1/2},$$

where 3^3/2^ is a conversion factor between shear stress and hardness. The values of Δ*H*_*v*_ for the current oxygen-doped Ti–30Zr–14Nb and Ti–30Zr–26Nb MEAs were obtained from nanoindentation measurements. Young’s moduli of the Ti–30Zr–14Nb and Ti–30Zr–26Nb MEAs are 61.5 and 77.8 GPa, and the shear moduli were calculated to be 23.7 and 29.9 GPa, respectively. As such, the calculated values of the tetragonal distortion Δ*ε* were determined to be 0.150, 0.171, 0.176, 0.180 and 0.193 for the Ti–30Zr–14Nb–*y*O, and 0.10, 0.147, 0.154, 0.159 and 0.161 for Ti–30Zr–26Nb–*y*O (*y* = 1, 2, 3, 3.5, and 4 at.%), respectively. These values are comparable to those of other tetragonal lattice distortions at room temperature^15,36^. Therefore, alloying oxygen enhances the asymmetry of tetragonal distortions, leading to a larger Δ*ε* value and hence more pronounced interstitial strengthening. In other words, the increase in the yield strength is mainly resulted from the interstitial solid-solution hardening of O.

**Fig. 5:**
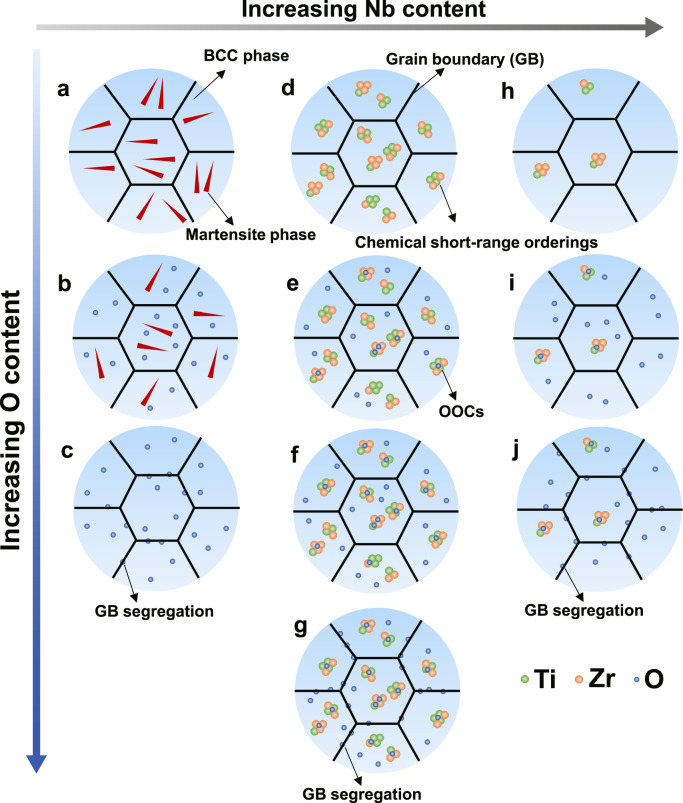
Schematic diagram for manipulating the OOCs in alloys. (**a**–**c**, **d**–**g**, and **h–j**) show the variation of OOC formation as a function of oxygen in the alloys with a low, optimal, and excessive Nb content, respectively. As shown, the optimal distribution of OOCs can be obtained by controlling Nb and oxygen contents. GB represents grain boundary, whilst green, orange, and blue bubbles represent Ti, Zr, and O atoms, respectively.

To further illustrate how to boost the OOC-mediated interstitial strengthening effect to improve the ductility of the current MEAs, a schematic diagram is shown in Fig. 5. In the dual-phase Ti–30Zr–10Nb MEA (Fig. 5a), i.e., in the alloys with a low amount of Nb, oxygen addition suppresses twinning and the martensite phase transformation (Fig. 5b), leading to the significant loss of ductility^9,18,19^. The addition of excess oxygen results in the grain boundary segregation (Fig. 5c), and then causes the catastrophic brittle fracture (Supplementary Fig. 1a). Nb is a bcc-phase stabilizer, and its increase promotes the formation of a single bcc phase structure in the oxygen-free base MEAs (Supplementary Fig. 2). Short-range orderings depend on the energetic competition between all possible phases at a fixed composition, and hence only coherent phase-separated states are of relevance for their formation^37^. Thus, coherent phase stability determines the short-range orderings in alloys. In the bcc alloy with a relatively low Nb content and correspondingly low phase stability, such as Ti–30Zr–14Nb MEA, numerous (Ti, Zr)-enriched CSROs exist in the matrix (Fig. 5d). Upon doping oxygen, oxygen atoms prefer to occupy interstitial positions adjacent to (Ti, Zr)-enriched lattice sites and thus form the (O, Ti, Zr)-enriched OOCs (Fig. 5e), as similarly reported in TiZrHfNb HEA^15^. Based on our experimental observations and DFT calculation results, we found that O atoms are more likely to occupy octahedral interstitial sites within (Ti, Zr)-CSROs in the current MEAs. In other words, when doping oxygen, the existence of (Ti, Zr)-enriched CSROs favors the formation of OOCs. In the Ti–30Zr–14Nb MEA, the number density of OOCs increases with the oxygen content (Fig. 5f), e.g., from 1.04 × 10^24^ m^-3^ for the Ti–30Zr–14Nb–1O MEA to 8.7 × 10^24^ m^-3^ for the Ti–30Zr–14Nb–3O variant. Because of insufficient OOCs in Ti–30Zr–14Nb–1O, the interaction between the OOCs and dislocations in this alloy is not that strong, and as a result, planar slip was dominant during deformation (Fig. 4g, h). With the increase of oxygen to 3 at.%, the number density of OOCs is increased, resulting in strong dislocation pinning effect and remarkably promoted cross-slips in Ti–30Zr–14Nb–3O. As such, the plastic deformation of the Ti–30Zr–14Nb–3O MEA is dominated by wavy slip (Fig. 4d–f). Such slip behavior efficiently suppresses the dislocation tangles and stress concentration of planar slips, then prevents the alloy from early fracture. Wavy dislocation configurations pronouncedly promote the dislocation nucleation and propagation, eventually enhancing the alloy’s work-hardening capacity and thereby its ductility^15^. Thus, increasing the amount of oxygen facilitates the OOC-mediated interstitial strengthening effect, yielding a larger ductility in Ti–30Zr–14Nb–3O MEA. However, too much oxygen doping would similarly cause grain boundary segregation (Fig. 5g), giving rise to the severe deterioration of ductility (Fig. 1a).

As the Nb content is increased, e.g., Ti–30Zr–26Nb MEA, the bcc phase becomes more stable and the (Ti, Zr)-enriched CSROs in the matrix (Fig. 5h) become less due to the reduced concentration of Ti (Fig. 2a–g). At the same level of oxygen, the number density of the formed OOCs decreases (Fig. 5e, i). For example, Ti–30Zr–26Nb–3O only has an OOC number density of 6.7 × 10^23^ m^-3^, much less than that of Ti–30Zr–14Nb–3O (i.e., 8.7 × 10^24^ m^-3^). The deformation in the former alloy is dominated by planar slip at the initial stage and wavy slip at the late stage. Planar slip is topologically confined and localized to some limited slip planes, leading to in-plane softening and high pile-up stresses, promoting damage initiation^38^. Thus, the OOC-mediated interstitial strengthening effect is much demoted, resulting in a great loss of ductility. Additionally, no oxygen segregation at grain boundaries was observed for the Ti–30Zr–14Nb–3O (Fig. 5f and Supplementary Fig. 13a, b) and Ti–30Zr–30Nb–2O MEAs (Fig. 5i and Supplementary Fig. 13c, d). Nevertheless, O segregation at grain boundaries did occur in Ti–30Zr–14Nb–4O (Figs. 3k1, k2, 5g) and Ti–30Zr–30Nb–3O MEAs (Figs. 3f1, f2, 5j), despite that no oxide was formed in both MEAs (Supplementary Fig. 14a, b). As a result, the fracture mode changed from ductile fracture (predominantly dimpled rupture) to intergranular brittle fracture mode in these two MEAs (Supplementary Fig. 14c–f). Therefore, the solid solubility of O atoms in the current MEAs decreases with the increase of Nb, which is consistent with the DFT calculations that the O solubility for Ti-30Zr-14Nb is higher than that for Ti-30Zr-30Nb MEA. Consequently, grain boundary embrittlement resulted from oxygen segregation is responsible for the premature fracture behavior and low ductility in the Ti-30Zr-14Nb-4O and Ti-30Zr-30Nb-3O MEAs.

Our approach is based on the creation of OOCs via promoting the formation of (Ti, Zr)-enriched CSROs, and the unique interstitial strengthening effects from OOCs simultaneously realized strengthening and ductilizing. Importantly, such a strategy can be readily extended to the alloy systems that can dissolve a certain amount of oxygen, such as Zr-based and Ti-based conventional alloys, and Zr–Ti–Nb–*M* (*M* = other metals) HEAs (Supplementary Fig. 15), demonstrating potential application prospects of the OOC-mediated interstitial strengthening effect.

In summary, we decipher the controlling factors for forming OOCs and elaborate the alloy design principle for the OOC-mediated interstitial strengthening effect. We find that, in the single bcc Ti–30Zr–*x*Nb (*x* = 14, 18, 22, 26, and 30 at.%) MEAs, the degree of CSROs increases by decreasing the bcc phase stability (i.e., reducing the Nb content). At the given Nb content, the number density of OOCs rises with doping oxygen, but excess oxygen addition tends to induce grain boundary segregation. At the given oxygen content, the decreased degree of CSROs caused by the increment in Nb demotes the formation of OOCs. Upon deformation, the enhanced number density of OOCs favors wavy slip, and promotes double cross-slip and thus dislocation multiplication, resulting in enhanced ductility. Meanwhile, the improved yield strength with the increase of O originates from the interstitial solid-solution hardening. The optimal OOC-mediated interstitial strengthening effect is observed in the Ti–30Zr–14Nb–3O MEA, whose tensile yield strength and ductility were improved by ~75% and ~164%, respectively. Our findings not only validate that this unique interstitial strengthening effect can be applied to alloy systems capable of dissolving a certain amount of oxygen, but also establish alloy design principles for the OOC formation, providing a paradigm for developing advanced alloys.

## Methods

### Materials preparation

Ingots with a nominal composition of Ti–30Zr–*x*Nb–*y*O (*x* = 10, 14, 18, 22, 26, and 30 at.%, and *y* = 0, 1, 2, 3, 3.5 and 4 at.%) MEAs were prepared by arc-melting a mixture of pure metals (purity larger than 99.95 wt.%), TiO_2_ (99.99 wt.%) in a Ti-gettered high-purity argon atmosphere. Supplementary Table 4 presents the inert gas fusion analysis results of oxygen in the as-cast MEAs. The actual oxygen content is slightly higher than the nominal one because of the unavoidable oxygen contamination during the melting process. Nevertheless, the trend in the actual oxygen content is consistent with that of the nominal oxygen value. The ingots were remelted at least six times to ensure chemical homogeneity, and then drop-cast into a water-cooled copper mold with a dimension of 10 × 10 × 60 mm^3^.

### Mechanical characterization

Room-temperature tensile tests were performed on a CMT4105 universal electronic tensile machine at a strain rate of 1 × 10^−3^ s^−1^. Dog bone-shaped tensile samples of each alloy with a gauge length of 20 mm and a cross-section of 1.3 × 5.0 mm^2^ were cut by electrical discharging. Before deformation, all tensile samples were polished down to the 2000-grit SiC paper.

### Structure and compositional characterization

Phase and dislocation characterization of the as-cast and the deformed alloys were conducted by X-ray diffractometer with Cu-Kα radiation (MXP21VAHF) and transmission electron microscope (TEM) with an FEI Tecnai G2 F30, respectively. An aberration-corrected FEI Titan Themis G2 with spatial resolutions up to 60 pm was used to analyze atomic structure of the as-cast alloys. HAADF and iDPC images were recorded at 300 kV. The convergence semi-angle for imaging is 30 mrad, and the collection semi-angle snap is 4 to 21 mrad for the iDPC imaging and 39 to 200 mrad for the HAADF. The iDPC and HAADF images were not from the same region, as they require different camera lengths and a slight change in focus and lens aberrations. Atomic strain maps were obtained using the geometric phase analysis method^39^. To minimize the possible influence of vibration during scanning on the strain maps, we took over 10 aberration-corrected TEM images of the same region and averaged the corresponding atomic positions. Although such atomic strain analysis is affected by camera resolution, qualitative difference in atomic strain maps between the Ti–30Zr–14Nb and Ti–30Zr–30Nb MEAs is substantial, indicating a large difference in lattice distortion between the two MEAs. For the TEM observation, specimens were new mechanically ground to 50 μm thickness, and then twin-jet electropolished using H_2_SO_4_ (10%) and CH_4_O (90%) solution under −30 °C.

Atom probe tomography and 3D elemental distribution analyses were carried out in CAMECA Instruments LEAP 5000XR local electrode atom probe system. The specimens were analyzed in voltage mode under an ultrahigh vacuum of approximately 2.5 × 10^−11^ Torr at 70 K, a target evaporation rate of 3 ions for 1000 pulses on average in high-voltage pulsing mode at 15% pulse fraction. Sharp tip specimens for the APT tests were prepared by focused ion beam milling on a dual-beam FEI Helios 600^40^. The CAMECA integrated visualization and analysis software IVAS 3.8.0 was used for data processing and three-dimensional (3D) atomic reconstruction.

### Internal-friction measurements

Beam-shaped samples of 1.0 × 2.0 × 50 mm (thickness × width × length) were used for damping-capacity measurement on a multifunction internal friction apparatus (MFP-1000) at low frequencies of 0.5, 1.0, 2.0, and 4 Hz. All the samples were polished down to a 2000-grit SiC paper to eliminate surface scratches. A continuous heating rate of 2 K min^-1^ from 300 to 1100 K in a vacuum and a forced vibration with the maximum strain amplitude of 2 × 10^-4^ were applied for the damping measurements. The background was subtracted using the following formula:

4 $${Q}_{b}^{-1}=A+B\exp \left(\frac{-C}{{k}_{B}T}\right),$$

where ${Q}_{b}^{-1}$ is the energy dissipation coefficient of the background, A, B, and C are the parameters to be determined after optimization of the *χ*^2^ function, *k*_*B*_ is the Boltzmann constant, and *T* is the temperature^41^. The fitting curves are in good agreement with the experimental data, and the correlation indexes are above 0.999.

### Theoretical calculations

The Vienna ab initio Simulation Package (VASP) was used to perform the density functional theory (DFT) calculations^42^. The electronic structures were described by Perdew, Burke and Ernzerhof (PBE)^43^ functional of generalized gradient approximation with projector augmented wave (PAW)^44^. The plane-wave basis kinetic energy cut-off was set to 400 eV. All atoms and the simulation cells were permitted to relax during the geometrical optimizations, then the lattice distortion could be taken into account when calculating the O solution energies. For the electronic structure optimization, the total energy was converged to less than 10^−6^ eV. To calculate the O solution energy, the maximum force on atoms less than 0.02 eV/atom was chosen as the convergence criterion for geometric optimization. The atomic structure of the lattice was first relaxed, and then O atoms were placed at the octahedral interstitial sites in the lattice for further relaxation.

BCC supercells of the size 4 × 2 × 6 with a total of 96 atoms were built to represent the Ti-30Zr-14Nb and Ti-30Zr-30Nb MEAs. Originally, the atoms of different elements were distributed randomly with no local chemical ordering. Then the Monte Carlo (MC) swap of atoms of different elements was performed to search for atomic configurations with lower system energy. If the energy of the new system is lower than the previous system, then accept the new atomic arrangement, otherwise accept the new system with a probability of $\exp (\frac{{\Delta}{E}}{{k}_{B}T})$, where Δ*E* is the calculated energy difference between the previous system and the new system energy, *k*_*B*_ is the Boltzmann constant, and *T* is the temperature. Here we adopted *T* = 300 K. The convergence criterion for the MC swap process is defined as the energy variance within 100 steps less than 0.01 eV/atom.

Then the models with the lowest system energy were selected during the MC swap processes as the matrix for calculating O solution energy in Ti–Zr–Nb MEAs. The O solution energies are calculated as

5 $${E}_{{{{{{\rm{O-solu}}}}}}}={E}_{{{{{{\rm{O+matrix}}}}}}}-{E}_{{{{{{\rm{matrix}}}}}}}-{E}_{O},$$

where *E*_O-solu_ is the total energy of the relaxed matrix with an O atom embedded in the matrix, *E*_matrix_ is the energy of the relaxed matrix, *E*_O_ is the energy of an O atom in a vacuum. Moreover, the equation for calculating the O solubility in the two models is^45^

6 $${C}_{O}=\int _{-\infty }^{+\infty }\frac{n({E}_{{{{{{\rm{O-solu}}}}}}})d{E}_{{{{{{\rm{O-solu}}}}}}}}{1+\exp (\frac{{E}_{{{{{{\rm{O-solu}}}}}}}-\mu }{{k}_{{{{{{\rm{B}}}}}}}T})},$$

where *n*(*E*_*O-solu*_) is the distribution of O solution energies, *μ* is the chemical potential of an O atom in the gas phase and can be calculated as $1/2{\mu }_{{O}_{2}}^{gas}$. Here we adopted a ${\mu }_{{{{{{{\rm{O}}}}}}}_{2}}^{{{{{{\rm{gas}}}}}}}=-9.99\,{{{{{\rm{eV}}}}}}$ at 1 bar and 300 K.

Ab initio molecular dynamics (AIMD) simulations were implemented for the bcc Ti–30Zr–14Nb and Ti–30Zr–30Nb MEAs. All calculations were performed by the Vienna ab initio simulation package (VASP). The 128 atoms supercells of each MEA were first generated with the special quasi-random structure (SQS) model as the initial configurations^46^. The systems were then melted and equilibrated at 1.5 times the melting point (*T*_*m*_) for 20 ps with a time step of 5 fs, and quenched to 300 K with a constant temperature, constant volume (NVT) ensemble^47^. The values of *T*_*m*_ used for the Ti–30Zr–14Nb and Ti–30Zr–30Nb MEAs are the theoretical values of 2110 K and 2240 K, respectively. Brillouin zone integrations were performed by using a single Γ-center k-point. The Warren-Cowley chemical short-range order parameter^48^ is defined as

7 $${\alpha }_{ij}=1-{N}_{ij}/N{X}_{j},$$

where *N*_*ij*_ is the number of *j*-type atoms in the first nearest neighboring around an *i*-type atom, *N* is the total number of atoms in the first nearest neighbor around an atom and *X*_*j*_ is the atomic fraction of type *j* in the alloy.

## Supplementary information

Supplementary Information

## Data Availability

The datasets generated and/or analyzed during the current study are available from the corresponding authors on request.

## References

[1] Tyson WR (1967). Strengthening of hcp Zr, Ti and Hf by interstitial solutes-a review. Can. Metall. Q..

[2] Conrad H (1981). Effect of interstitial solutes on the strength and ductility of titanium. Prog. Mater. Sci..

[3] Yu Q (2015). Origin of dramatic oxygen solute strengthening effect in titanium. Science.

[4] Yang PJ (2019). Mechanism of hardening and damage initiation in oxygen embrittlement of body-centred-cubic niobium. Acta Mater..

[5] Jo MG, Madakashira PP, Suh JY, Han HN (2016). Effect of oxygen and nitrogen on microstructure and mechanical properties of vanadium. Mater. Sci. Eng. A.

[6] Roumina R, Embury JD, Bouaziz O, Zurob HS (2013). Mechanical behavior of a compositionally graded 300M steel. Mater. Sci. Eng. A.

[7] Svoboda J, Ecker W, Razumovskiy VI, Zickler GA, Fischer FD (2019). Kinetics of interaction of impurity interstitials with dislocations revisited. Prog. Mater. Sci..

[8] Courtney, T. H. *Mechanical Behavior of Materials* (Waveland Press, 2005).

[9] Wei QQ (2011). Influence of oxygen content on microstructure and mechanical properties of Ti-Nb-Ta-Zr alloy. Mater. Des..

[10] Ando T, Nakashima K, Tsuchiyama T, Takaki S (2008). Microstructure and mechanical properties of a high nitrogen titanium alloy. Mater. Sci. Eng. A.

[11] Nakada Y, Keh AS (1971). Solid-solution strengthening in Ni-C alloys. Metall. Trans..

[12] Nakada Y, Keh AS (1968). Solid solution strengthening in Fe-N single crystals. Acta Met..

[13] Dadfarnia M (2010). Recent advances in the study of structural materials compatibility with hydrogen. Adv. Mater..

[14] Somerday, B., Sofronis, P. & Jones, R. H. Effects of hydrogen on materials. In: *Proceedings of the 2008 International Hydrogen Conference*, Jackson Lake Lodge, Grand Teton National Park, Wyoming, USA (ASM International, 2009).

[15] Lei Z (2018). Enhanced strength and ductility in a high-entropy alloy via ordered oxygen complexes. Nature.

[16] Lei Z (2020). Snoek-type damping performance in strong and ductile high-entropy alloys. Sci. Adv..

[17] Cottrell AH, Bilby BA (1949). Dislocation theory of yielding and strain ageing of iron. Proc. Phys. Soc. A..

[18] Li Q (2018). Low Young’s modulus Ti-Nb-O with high strength and good plasticity. Mater. Trans..

[19] Besse M, Castany P, Gloriant T (2011). Mechanisms of deformation in gum metal TNTZ-O and TNTZ titanium alloys: a comparative study on the oxygen influence. Acta Mater..

[20] Pennycook SJ, Rafferty B, Nellist PD (2000). Z-contrast imaging in an aberration-corrected scanning transmission electron microscope. Microsc. Microanal..

[21] Bu YQ (2021). Local chemical fluctuation mediated ductility in body-centered-cubic high-entropy alloys. Mater. Today.

[22] Lazić I, Bosch EGT, Lazar S, Wirix M, Yücelen E (2016). Integrated differential phase contrast (iDPC)-direct phase imaging in STEM for thin samples. Microsc. Microanal..

[23] Lazić I, Bosch EGT (2017). Analytical review of direct stem imaging techniques for thin samples. Adv. Imaging Electron Phys..

[24] Lazić I, Bosch EGT, Lazar S (2017). Integrated differential phase contrast (iDPC) STEM. Acta Crystallogr..

[25] Zhang Y (2019). Effect of oxygen interstitial ordering on multiple order parameters in rare earth ferrite. Phys. Rev. Lett..

[26] Yucelen E, Lazic I, Bosch EGT (2018). Phase contrast scanning transmission electron microscopy imaging of light and heavy atoms at the limit of contrast and resolution. Sci. Rep..

[27] Reimer, L. *Transmission Electron Microscopy: Physics of Image Formation and Microanalysis* (Springer, 2013).

[28] Mughrabi, H., Ackermann, F. & Herz, K. *Fatigue Mechanisms* ASTM STP 675, 69 (American Society for Testing Materials, 1979).

[29] Bader, R. F.W. *Atoms in Molecule: A Quantum Theory* (Oxford University Press, 1990).

[30] Petch NJ (1953). The cleavage strength of polycrystals. J. Iron Steel I.

[31] Hall EO (1951). The deformation and ageing of mild steel: III. Discussion of results. Proc. Phy. Soc. B.

[32] Cordero ZC, Knight BE, Schuh CA (2016). Six decades of the Hall-Petch effect-a survey of grain-size strengthening studies on pure metals. Int. Mater. Rev..

[33] Fleischer RL (1962). Rapid solution hardening, dislocation mobility, and the flow stress of crystals. J. Appl. Phys..

[34] Courtney, T. H. *Mechanical Behaviour of Materials* (Waveland Press, 2005).

[35] Schuh CA, Nieh TG, Iwasaki H (2003). The effect of solid solution W additions on the mechanical properties of nanocrystalline Ni. Acta Mater..

[36] Mitchell TE, Heuer AH (1977). Solution hardening by aliovalent cations in ionic crystals. Mater. Sci. Eng. A.

[37] Wolverton C, Ozolins V, Zunger A (2000). Short-range-order types in binary alloys: a reflection of coherent phase stability. J. Phys.: Condens. Matter.

[38] Gerold V, Karnthaler HP (1989). On the origin of planar slip in FCC alloys. Acta Met..

[39] Hÿtch MJ, Snoeck E, Kilaas R (1998). Quantitative measurement of displacement and strain fields from HREM micrographs. Ultramicroscopy.

[40] Thompson K (2007). In situ site-specific specimen preparation for atom probe tomography. Ultramicroscopy.

[41] Nowick, A. S. *Anelastic Relaxation in Crystalline Solids* Vol.1 (Elsevier, 2012**)**.

[42] Kresse G, Furthmuller J (1996). Efficient iterative schemes for ab initio total-energy calculations using a plane-wave basis set. Phys. Rev. B.

[43] Perdew JP, Burke K, Ernzerhof M (1996). Generalized gradient approximation made simple. Phys. Rev. Lett..

[44] Kresse G, Joubert D (1999). From ultrasoft pseudopotentials to the projector augmented-wave method. Phys. Rev. B.

[45] Zhou X, Curtin WA (2020). First principles study of the effect of hydrogen in austenitic stainless steels and high entropy alloys. Acta Mater..

[46] Zunger A, Wei S, Ferreira LG, Bernard JE (1990). Special quasirandom structures. Phys. Rev. Lett..

[47] Nosé S (1984). A unified formulation of the constant temperature molecular dynamics methods. Chem. Phys..

[48] Cowley JM (1950). An approximate theory of order in alloys. Phys. Rev..

