# Supplementary material for: Manipulating the ordered oxygen complexes to achieve high strength and ductility in medium-entropy alloys
Source: Nat Commun. 2023 Feb 13;14:806. doi: 10.1038/s41467-023-36319-0 (PMC9925791; doi:10.1038/s41467-023-36319-0)
Supplement: Supplementary file 1 — Supplementary Information [file 41467_2023_36319_MOESM1_ESM.pdf]

Supplementary Information for:

Manipulating the ordered oxygen complexes to achieve high strength and ductility in medium-entropy alloys

Authors: Meiyuan Jiao[1], Zhifeng Lei[2,*], Yuan Wu[1,*], Jinlong Du[3], Xiao-Ye Zhou[4], Wenyue Li[1], Xiaoyuan Yuan[1], Xiaochun Liu[5], Xiangyu Zhu[6], Shudao Wang[1], Huihui Zhu[1], Peipei Cao[1], Xiongjun Liu[1], Xiaobin Zhang[1], Hui Wang[1], Suihe Jiang[1], Zhaoping Lu[1,*]

Affiliations:

[1]*Beijing Advanced Innovation Center for Materials Genome Engineering, State Key Laboratory for Advanced Metals and Materials, University of Science and Technology Beijing, Beijing, 100083, China*
[2]*College of Materials Science and Engineering, Hunan University, Changsha, 410082, China*
[3]*Electron Microscopy Laboratory, School of Physics, Peking University, Beijing 100871, China*
[4]*Guangdong Province Key Laboratory of Durability for Marine Civil Engineering, School of Civil Engineering, Shenzhen University, Shenzhen, 518060, China*
[5]*Institute of Metals, College of Materials Science and Engineering, Changsha University of Science & Technology, Changsha, 410114, China*
[6]*Department of Materials Science and Engineering, the University of Texas at Dallas, Richardson, TX 75080, United States*
*Corresponding to: zflei@hnu.edu.cn; wuyuan@ustb.edu.cn; luzp@ustb.edu.cn

Supplementary Discussion

(1) DFT calculations on the electronic origins of (Ti, Zr)-CSROs and OOCs

Supplementary Fig. 9 presents the results of DFT calculations of the electronic origins of (Ti, Zr)-CSROs and OOCs. Initially, all constituent atoms were distributed randomly with no local chemical ordering. Then, a Monte Carlo swap process for different atoms to search for atomic configurations with the lowest system energy. Supplementary Fig. 9a and 9b show the typical atomic structure of Ti-30Zr-14Nb and Ti-30Zr-30Nb MEAs before and after the Monte Carlo swap process, respectively. The

number of Ti-Nb and Zr-Nb bonds decreases from 87 to 73 for Ti-30Zr-14Nb MEA and from 172 to 115 for Ti-30Zr-30Nb MEA, respectively, indicating the tendency of Nb atoms prefer to bond with other Nb atoms, rather than Ti and Zr atoms. In addition, the sum of Ti-Ti, Ti-Zr and Zr-Zr bonds decreases from 490 for Ti-30Zr-14Nb to 225 for Ti-30Zr-30Nb, which is consistent with the results that the degree of (Ti, Zr)-CSROs decreased with the increase of Nb.

Subsequently, we selected the atomic configurations with the lowest system energy after the Monte Carlo swap process as the matrix for calculating O solution energy in Ti-Zr-Nb MEAs. We found that the O solution energies at octahedral interstitial sites are lower than those at tetrahedral interstitial sites. Then, the local atomic environments of the O atoms at the octahedral interstitial sites in the Ti-30Zr-14Nb MEA were summarized by identifying the 6 nearest neighbors of the embedded O atoms (Supplementary Fig. 9c). The O solution energy decreases with increasing Ti and Zr atoms occupying the nearest neighbors, while increases with increasing Nb atoms. Hence, oxygen atoms are more likely to occupy octahedral interstitial sites in the (Ti, Zr)-CSROs, leading to the formation of OOCs. Moreover, O solution energies at all the 200 octahedral interstitial sites of the Ti-30Zr-14Nb and Ti-30Zr-30Nb MEAs were calculated. The distribution of O solution energies of the two modeled MEAs is shown in Supplementary Fig. 9d. Clearly, the O solution energies in Ti-30Zr-30Nb MEA are higher than that in Ti-30Zr-14Nb MEA. Therefore, a lower Nb composition in Ti-30Zr-14Nb MEA renders more solution sites for O than that in higher-Nb containing Ti-30Zr-30Nb MEA, resulting in a higher number density of OOCs when doping the same amount of oxygen.

(2) Bonding properties of the Ti-30Zr-14Nb and oxygen-doped Ti-30Zr-14Nb MEAs

Bonding properties of the Ti-30Zr-14Nb and oxygen-doped Ti-30Zr-14Nb MEAs were investigated by conducting the Bader charge analysis[1]. Bader charge can quantitatively characterize the charge transfer between atoms, and the results can be

found in Supplementary Table 2. Here, we studied the charge transfer (variation of the Bader charge) of metal atoms and O atoms at the two sites (see Supplementary Fig. 10) before and after the addition of O, respectively. One can see that after doping O atoms, Ti and Zr atoms lose more electrons while O atoms receive electrons in Site 1 (i.e., the interstitial site within Ti- and/or Zr-rich region), leading to an increase in the valence of Ti and Zr. In Site 2 (i.e., the interstitial site within Nb-rich region), nevertheless, Nb and O atoms both receive electrons, and Nb atoms receive fewer electrons after the addition of O atoms. Since the electronegativity of O is large, it is easier for O atoms to obtain the electrons from metal atoms with weak electronegativity (1.54 for Ti, 1.33 for Zr and 1.6 for Nb at the Pauling scale) to fill their outermost orbits and then reduce the energy. Therefore, O atoms prefer to bond with Ti and Zr atoms which are more likely to donate electrons to O atoms, rather than with Nb, and occupy the octahedral interstitial sites within (Ti, Zr)-CSROs to form OOCs.

Supplementary Figures

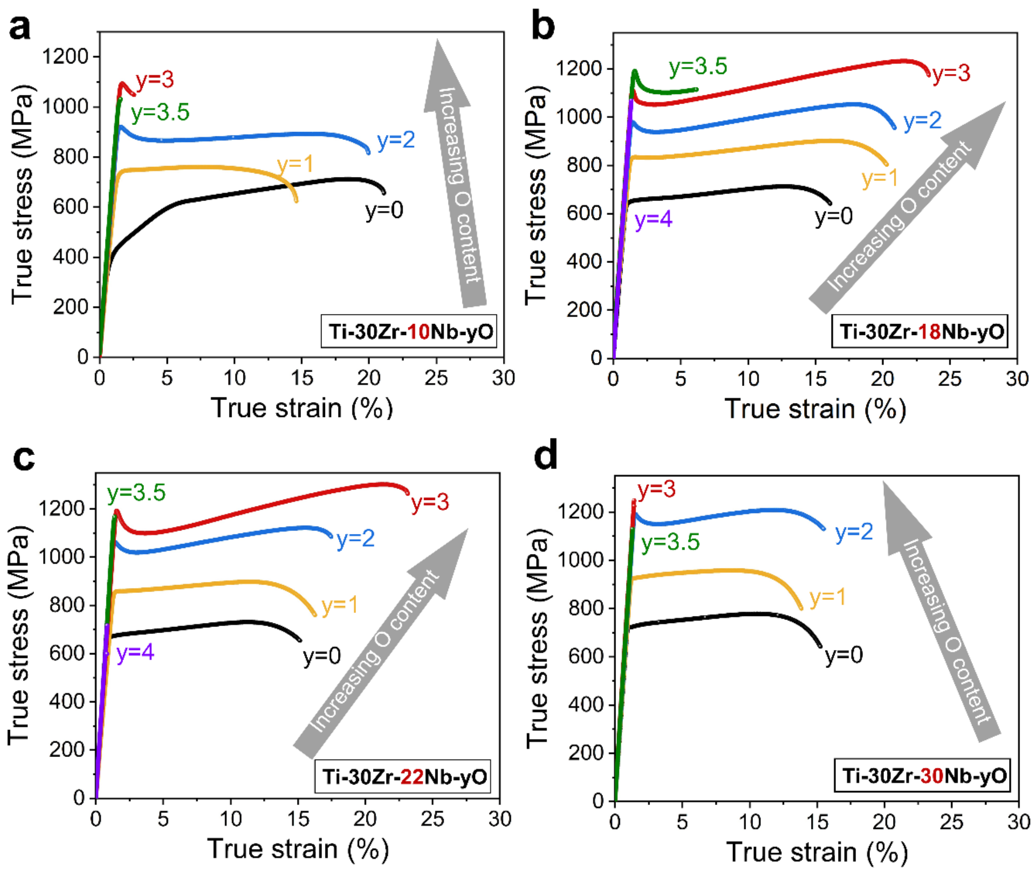

Supplementary Fig. 1 Tensile true stress-strain curves of the as-cast (a) Ti-30Zr-10Nb, (b) Ti-30Zr-18Nb, (c) Ti-30Zr-22Nb and (d) Ti-30Zr-30Nb MEAs added with a different amount of oxygen.

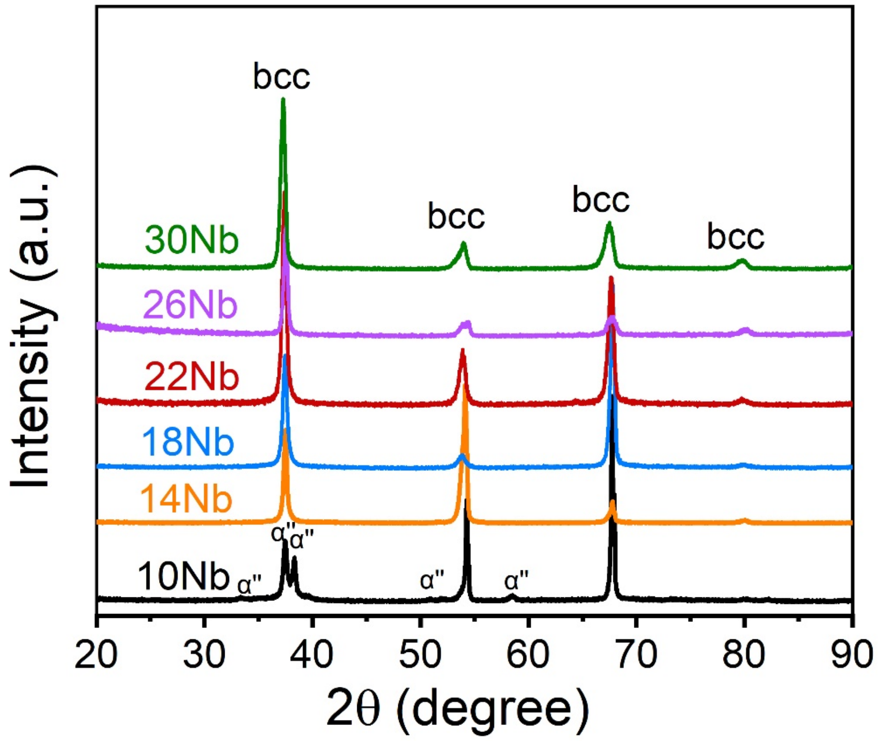

Supplementary Fig. 2 XRD patterns of Ti-30Zr-xNb (x = 10, 14, 18, 22, 26, and 30 at.%) MEAs.

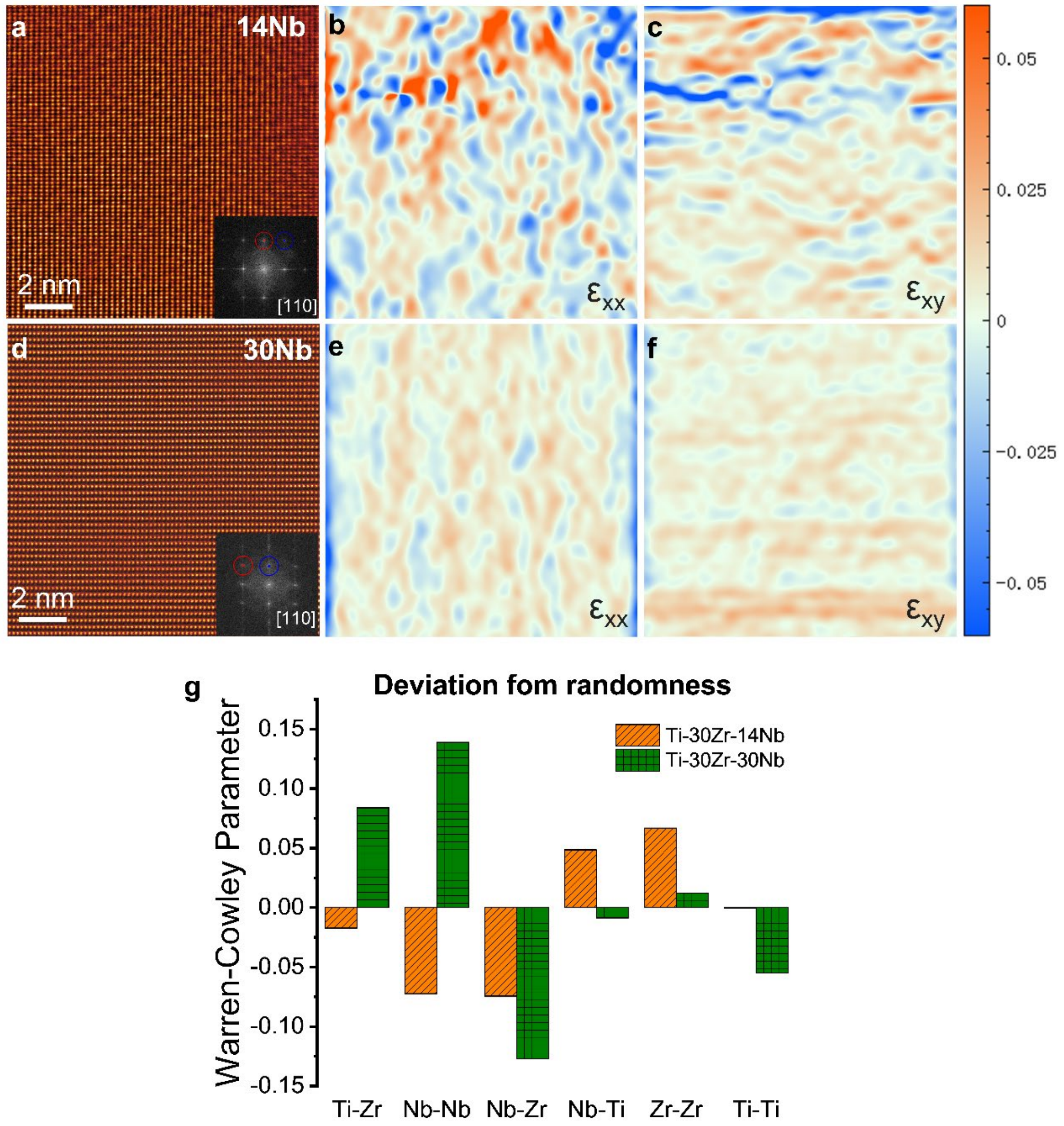

Supplementary Fig. 3 Comparison of atomic strain distribution between the (a-c) Ti-30Zr-14Nb and (d-f) Ti-30Zr-30Nb MEAs. (a,d) STEM-HAADF images of the Ti-30Zr-14Nb and Ti-30Zr-30Nb MEAs, respectively. The fast Fourier transformed images are shown in the insets. (b,c) Strain maps of (a) showing nanometer-sized local fluctuations of strain (ε_{xx}, horizontal normal strain; ε_{xy}, shear strain). (e,f) Strain maps of (d) show similar but much weaker contrast of local strains. The atomic strain field in the Ti-30Zr-30Nb MEA is more uniform, whereas substantial atomic strain fluctuations exist in the Ti-30Zr-14Nb MEA. (g) Warren-Cowley (WC) parameters for all atomic pairs in Ti-30Zr-14Nb and Ti-30Zr-30Nb. The value of the WC parameter of zero represents a purely random solid solution case, whereas negative values reflect the

favored atomic pairs and positive values indicate repulsive pairs. The value of Ti-Zr and Nb-Nb pairs is negative in Ti-30Zr-14Nb but positive in Ti-30Zr-30Nb, confirming the formation of the Ti-Zr and Nb-Nb pairs is more favored in the former.

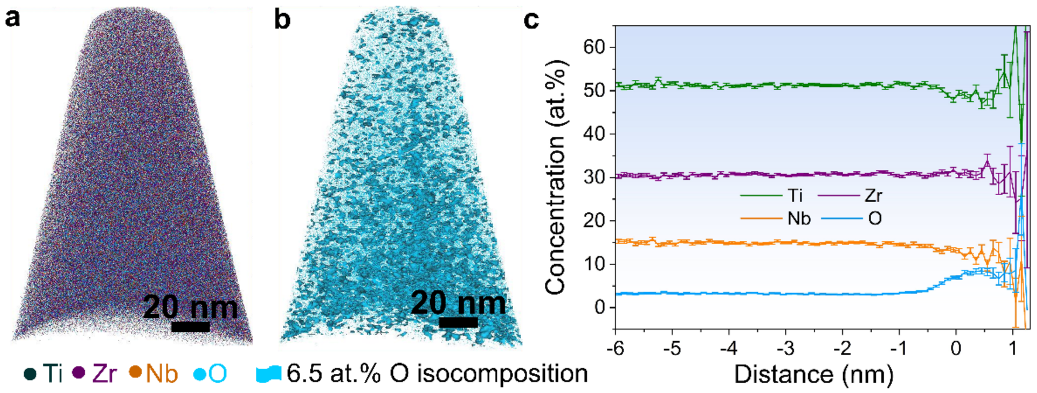

Supplementary Fig. 4 Elemental distribution in the Ti-30Zr-14Nb-3O MEA. (a) Three-dimensional reconstruction of the atomic distribution of Ti, Zr, Nb, and O in Ti-30Zr-14Nb-3O MEA. (b) The iso-composition surface with a threshold oxygen concentration of 6.5 at.%, highlighting the presence of OOCs. (c) Proximity analysis from the interface between the matrix/and OCCs indicates the enrichment of O, Ti, Zr and depletion of Nb.

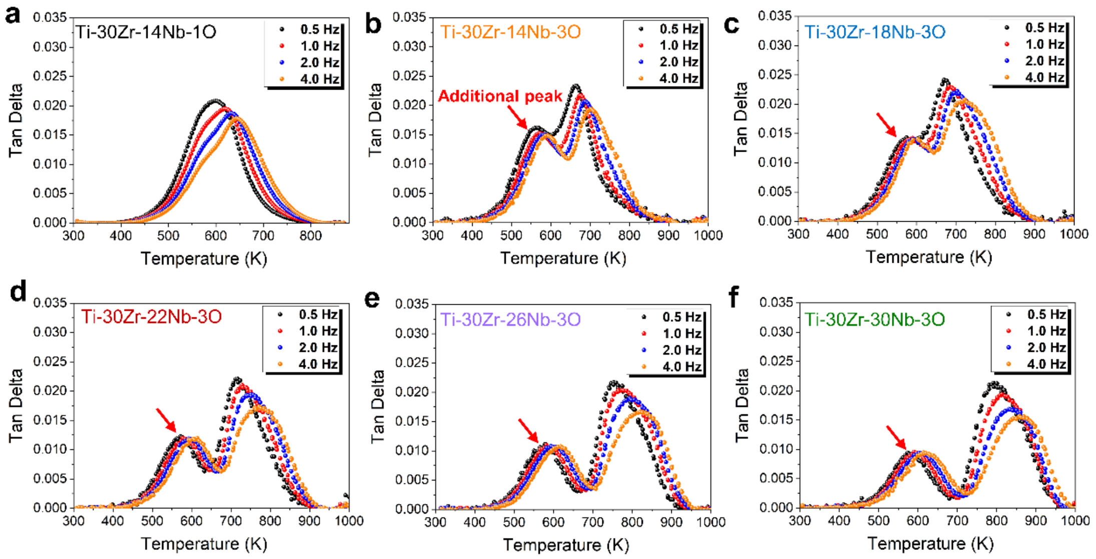

Supplementary Fig. 5 Temperature-dependent variation of damping capacity (indexed by Tan Delta) of the as-cast (a) Ti-30Zr-14Nb-1O, (b) Ti-30Zr-14Nb-3O, (c) Ti-30Zr-18Nb-3O, (d) Ti-30Zr-22Nb-3O, (e) Ti-30Zr-26Nb-3O and (f) Ti-30Zr-30Nb-3O MEAs. The measurements were conducted on a multifunction internal friction apparatus by the forced vibration at 0.5, 1.0, 2.0 and 4.0 Hz. The red arrows in the figures indicate the additional low-temperature peak which reflects the existence of OOCs.

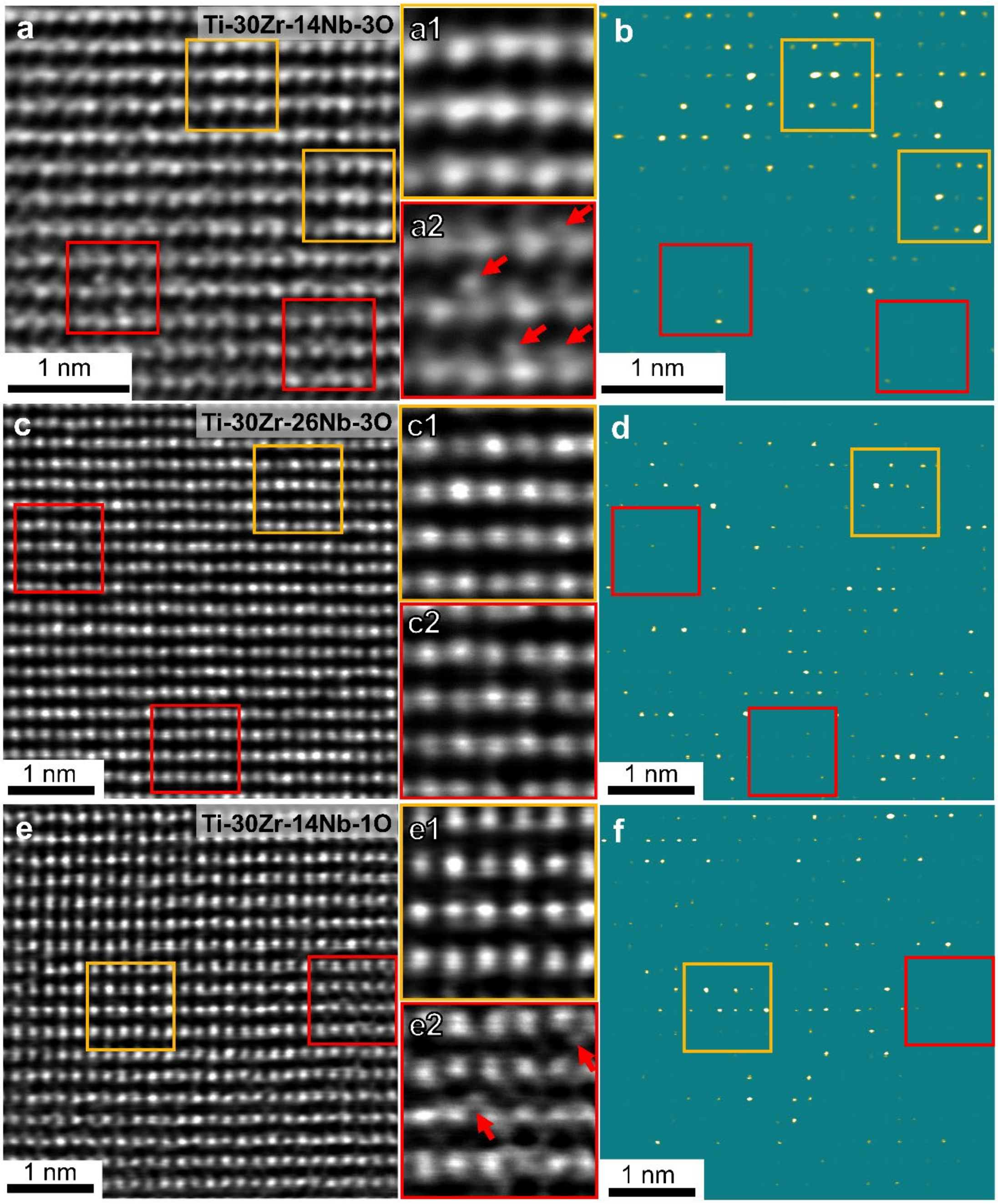

Supplementary Fig. 6 Ordered oxygen complexes (OOCs) in three MEAs. (a, b), (c, d) and (e, f) iDPC images taken from the $[011]_{bcc}$ zone axis with different contrasts to reveal the existence of chemical SROs in the Ti-30Zr-14Nb-3O, Ti-30Zr-26Nb-3O and Ti-30Zr-14Nb-1O MEAs, respectively. Red squares represent the Ti/Zr-rich regions and orange squares indicate the Nb-rich regions. (a1, a2), (c1, c2) and (e1, e2) Enlargements of the orange and red squares in a, c and e, respectively. Red arrows in a2 and e2 represent the existence of oxygen atomic columns, demonstrating the formation of

OOCs in Ti-30Zr-14Nb-3O and Ti-30Zr-14Nb-1O MEAs.

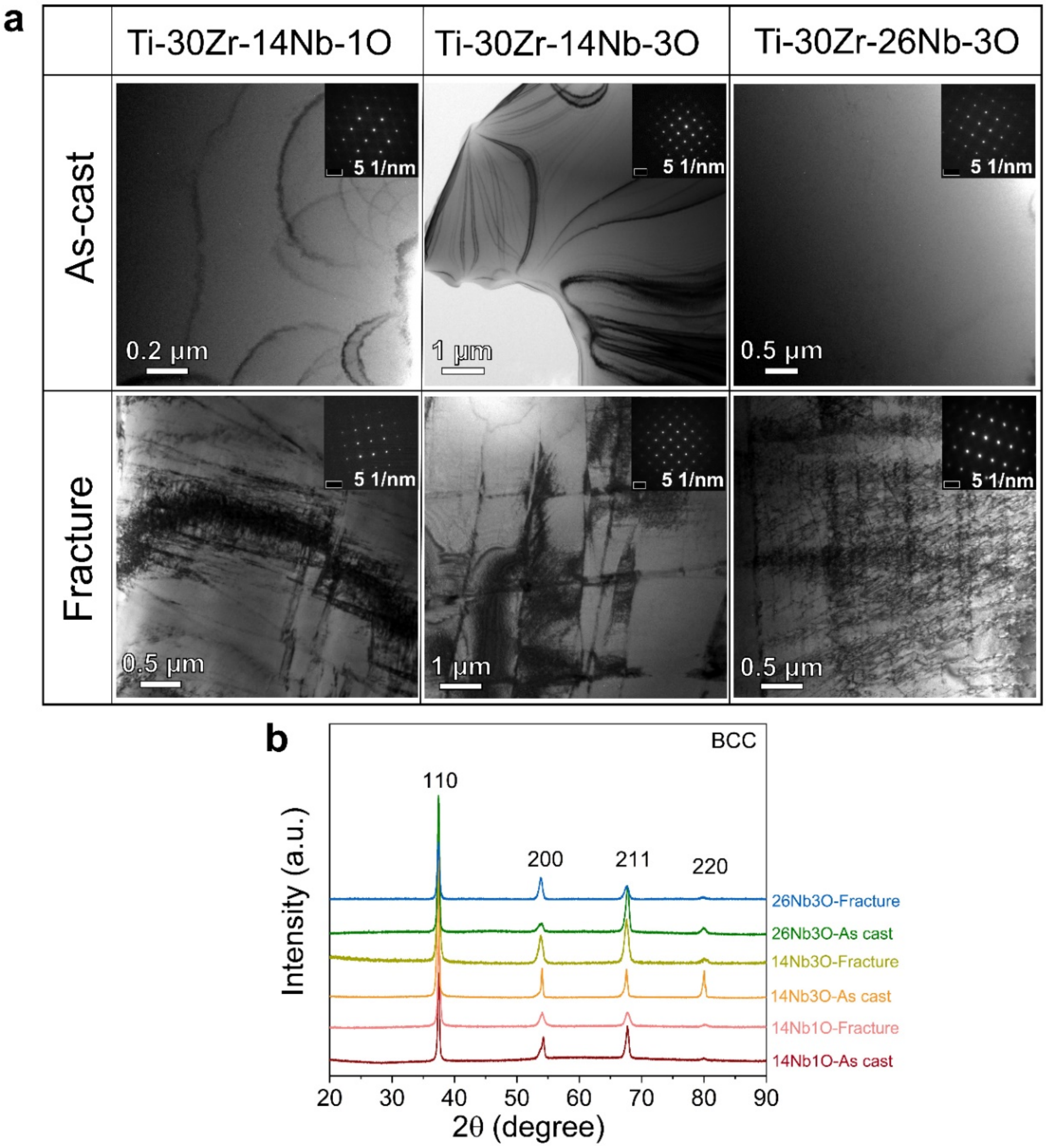

Supplementary Fig. 7 TEM images and XRD patterns of the as-cast and fractured samples of typical MEAs. (a) TEM images of the as-cast and fractured Ti-30Zr-14Nb-1O, Ti-30Zr-14Nb-3O, and Ti-30Zr-26Nb-3O MEAs, indicating that no second phase or twinning appears before and after the tensile tests. (b) XRD patterns of as-cast and fractured Ti-30Zr-14Nb-1O, Ti-30Zr-14Nb-3O, and Ti-30Zr-26Nb-3O MEAs further confirm that there is no phase transformation in three alloys during deformation.

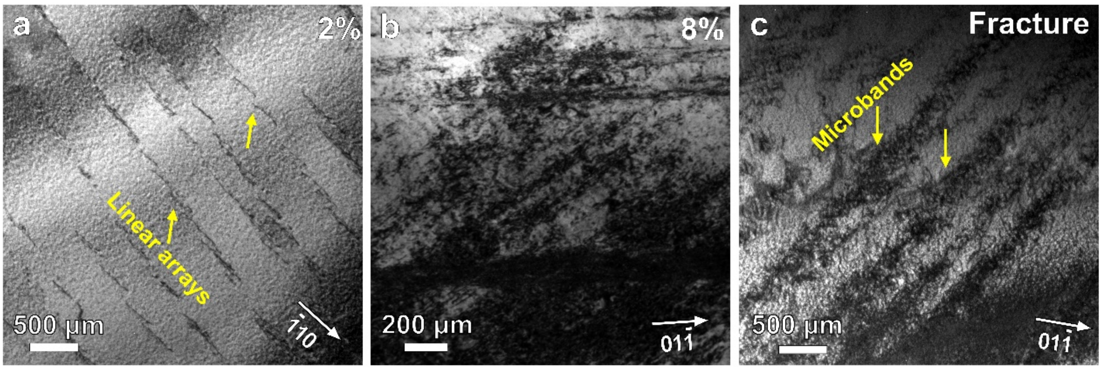

Supplementary Fig. 8 Bright-field TEM images of the Ti-30Zr-14Nb MEA pre-strained to (a) 2%, (b) 8% and (c) fracture, respectively. At the low tensile strain (i.e., 2%), dislocations in linear arrays (yellow arrows) were formed. As the strain increases to 8%, planar slip bands were observed. After fracture, microbands (yellow arrows) were clearly seen, indicating that planar slip is the dominant deformation mode.

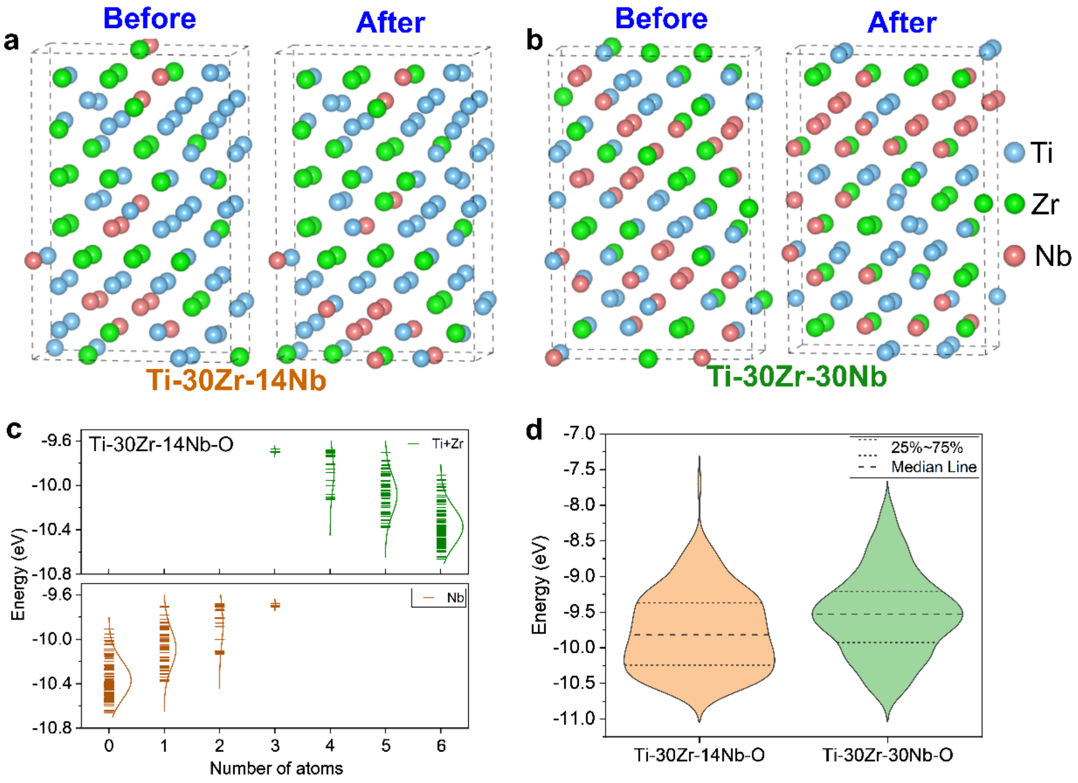

Supplementary Fig. 9 DFT calculations manifesting the electronic origins for forming (Ti, Zr)-CSROs and OOCs. (a,b) Atomic configurations of the Ti-30Zr-14Nb and Ti-30Zr-30Nb MEAs before and after the Monte Carlo swap process, respectively. Blue, green and pink bubbles represent Ti, Zr, Nb and O atoms, respectively. (c) The O solution energy against the number of atoms as the nearest neighbors in O-doped Ti-30Zr-14Nb MEA, indicating that the O solution energy decreases as more Ti and Zr atoms occupy the nearest neighbors. (d) Distribution of the O solution energies at all the 200 octahedral interstitial sites of Ti-30Zr-14Nb and Ti-30Zr-30Nb MEAs.

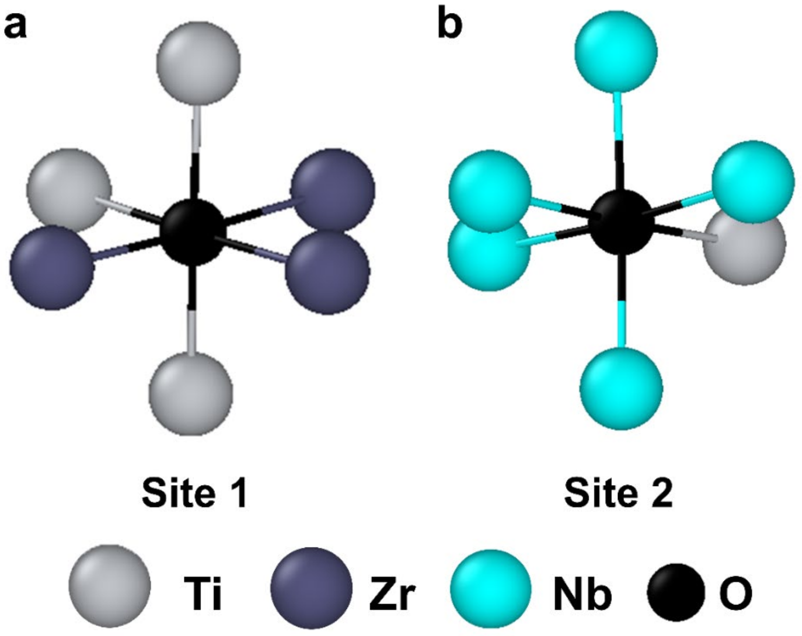

Supplementary Fig. 10 Electronic structures for (a) Site 1 (3Ti3Zr) and (b) Site 2 (1Ti5Nb) in O-doped Ti-30Zr-14Nb MEA. Grey, blue, cyan and black bubbles represent Ti, Zr, Nb and O atoms, respectively.

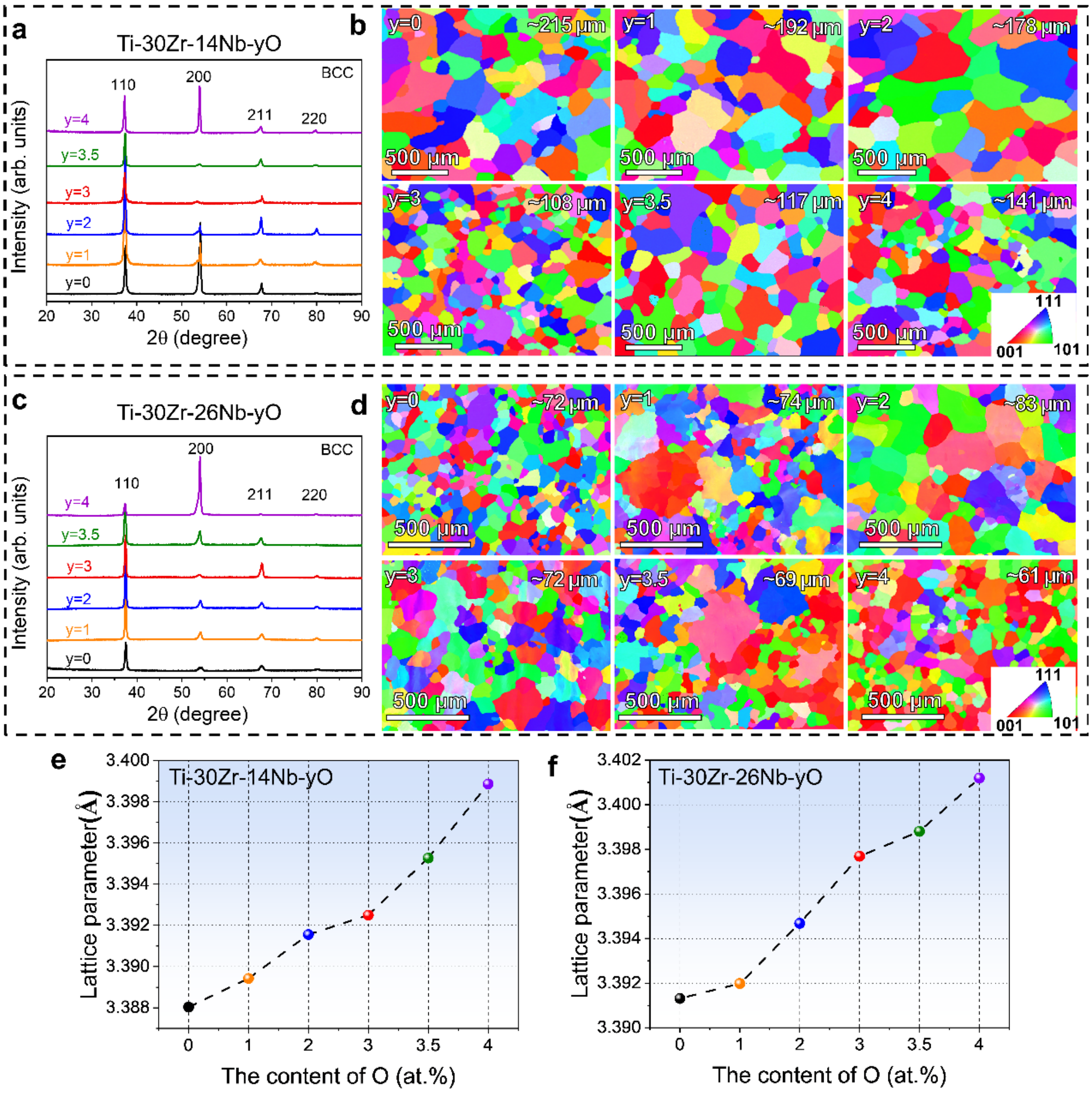

Supplementary Fig. 11 Microstructures of oxygen-doped Ti-30Zr-14Nb and Ti-30Zr-26Nb MEAs. (a, c) X-ray diffraction spectrums and (b, d) electron back-scattering diffraction (EBSD) patterns of the as-cast Ti-30Zr-14Nb-yO and Ti-30Zr-26Nb-yO (y = 0, 1.0, 2.0, 3.0, 3.5 and 4 at.%) MEAs, respectively, indicating that no second phase was detected. (e, f) The calculated lattice parameters of the Ti-30Zr-14Nb-yO and Ti-30Zr-26Nb-yO (y = 0, 1.0, 2.0, 3.0, 3.5 and 4 at.%) MEAs, respectively.

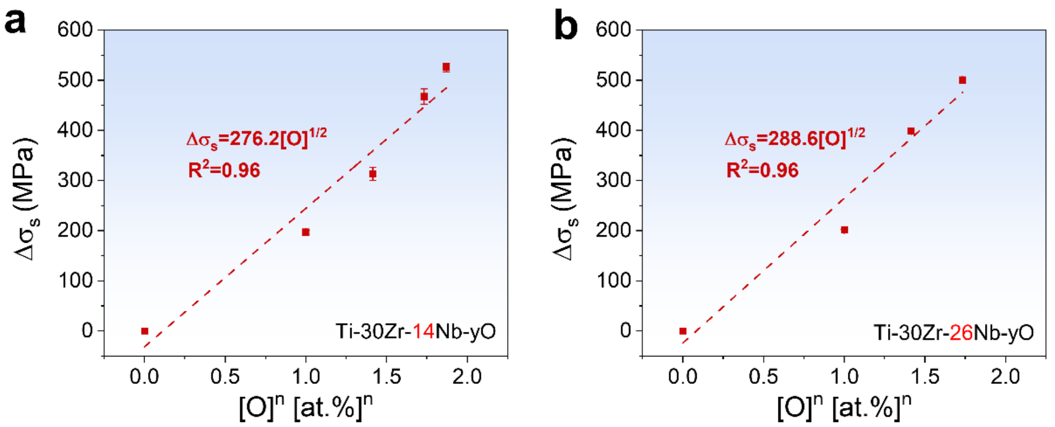

Supplementary Fig. 12 Variation of yield stress with the oxygen content in the (a) Ti-30Zr-14Nb, (b) Ti-30Zr-26Nb MEAs. The data were fitted by the Fleischer model. $\Delta\sigma_s$ represents the increase in the yield strength, $[O]^n$ represents the 1/2 power of the oxygen content, and R^2 is the fitting correlation index.

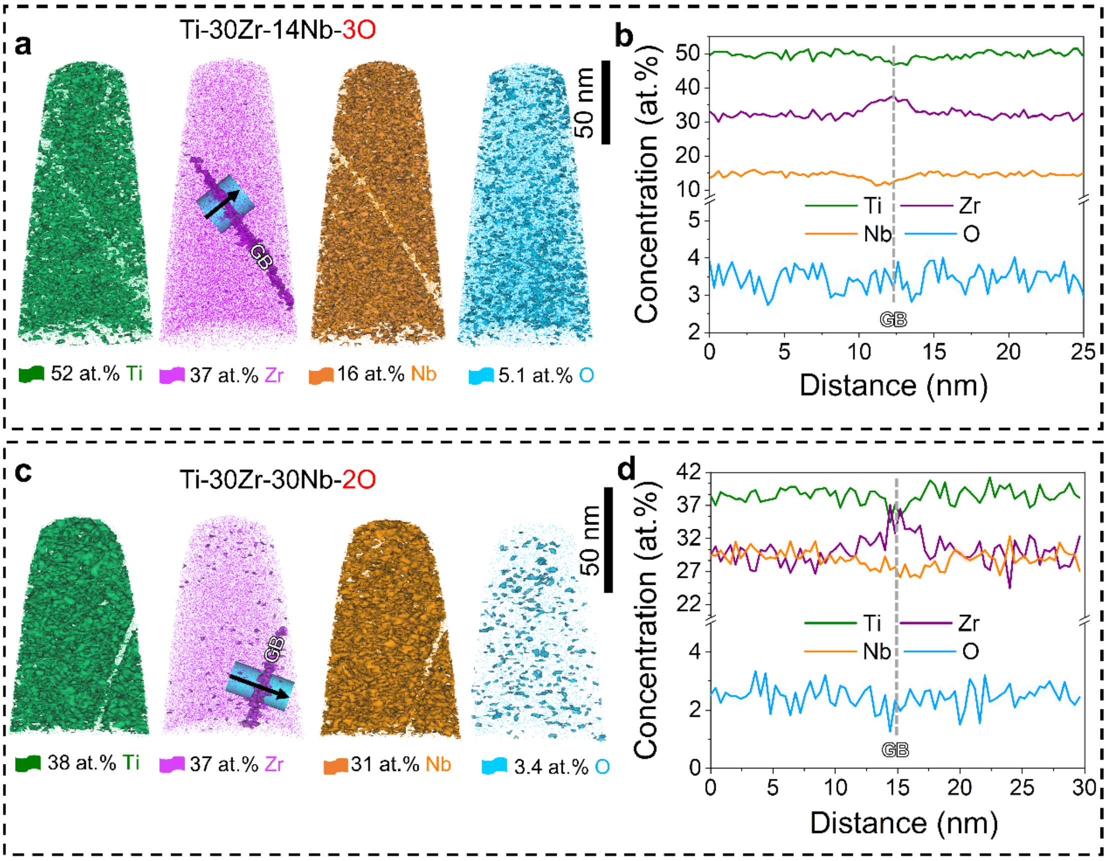

Supplementary Fig. 13 Elemental distribution in the as-cast Ti-30Zr-14Nb-3O and Ti-30Zr-30Nb-2O MEAs. (a, c) 3D APT tip reconstruction of the Ti-30Zr-14Nb-3O and Ti-30Zr-30Nb-2O MEAs, respectively. No oxygen segregates at grain boundaries (GB) in both MEAs. (b, d) The corresponding 1D concentration profiles across the grain boundaries (indicated by the black arrows in a and c), further indicate that no oxygen segregation at the grain boundaries.

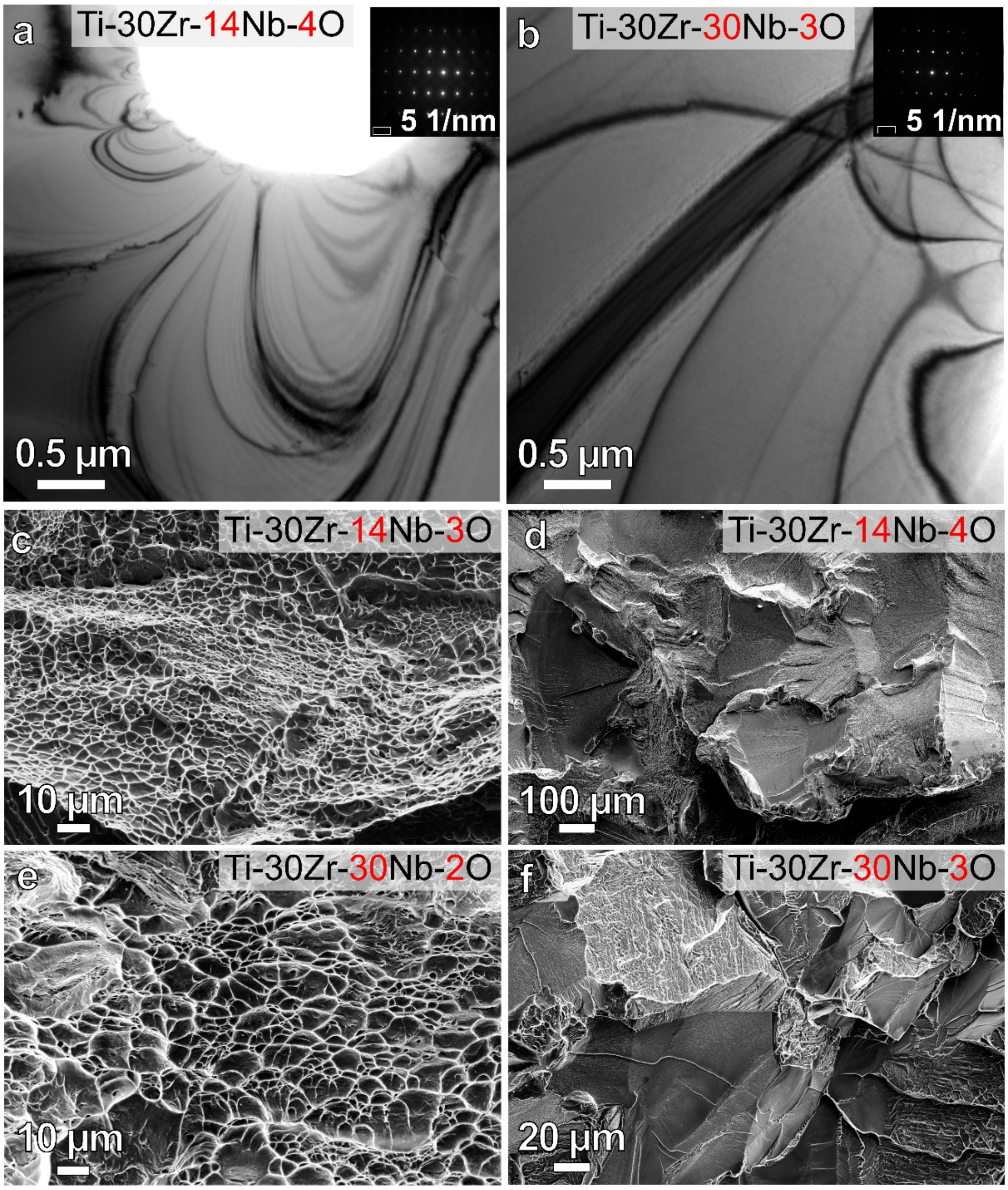

Supplementary Fig. 14 The as-cast microstructure and fracture tomography of the Ti-30Zr-14Nb and Ti-30Zr-30Nb MEAs doped with high contents of oxygen. (a, b) TEM images of the as-cast Ti-30Zr-14Nb-4O and Ti-30Zr-26Nb-3O MEAs, confirming that no oxide forms in both MEAs. (c-f) Fracture tomography of Ti-30Zr-14Nb-3O, Ti-30Zr-14Nb-4O, Ti-30Zr-30Nb-2O and Ti-30Zr-30Nb-3O MEAs, respectively. A gradual transition from ductile fracture (predominantly dimpled rupture) to intergranular brittle fracture mode was noticed with the increase of oxygen.

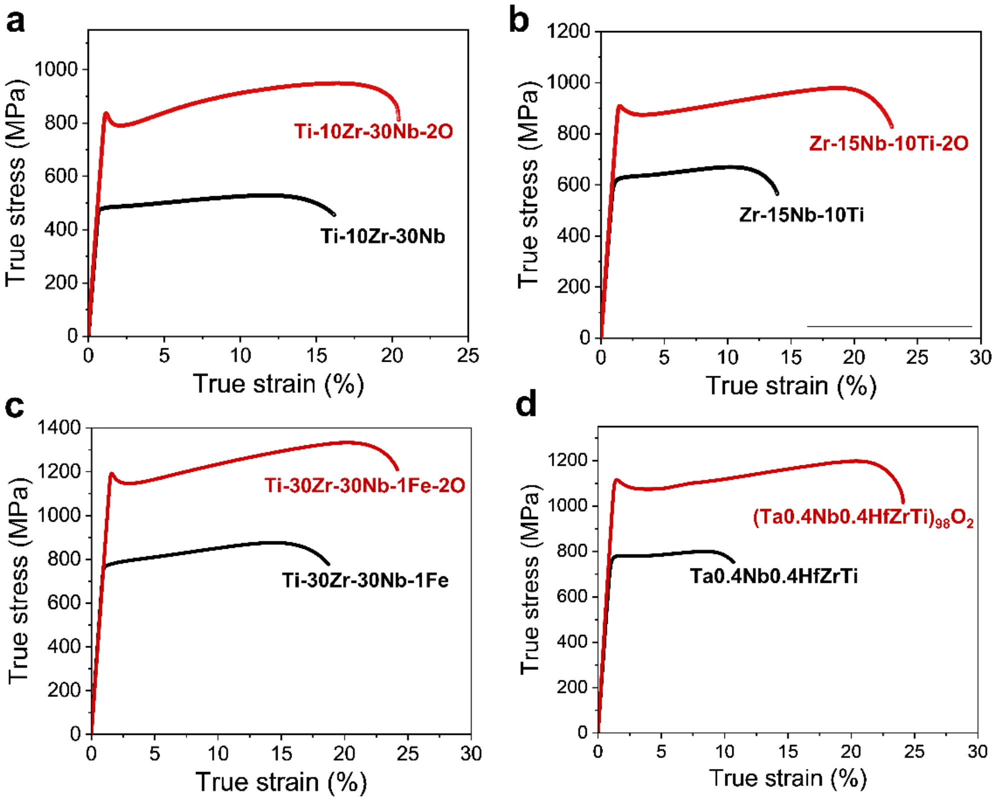

Supplementary Fig. 15 Room-temperature tensile true stress-strain curves of the as-cast (a) Ti-10Zr-30Nb, (b) Zr-15Nb-10Ti, (c) Ti-30Zr-30Nb-1Fe, (d) Ta0.4Nb0.4HfZrTi alloys doped with 2 at.% oxygen content. Results reveal that the mechanism of introducing ordered oxygen complex-mediated interstitial strengthening works for other alloys, such as Ti-based and Zr-based conventional alloys, and Zr-Ti-Nb-M (M=other metals) HEAs.

Supplementary Table 1 Cluster analysis parameters, d_{max} and N_{min}, used for the three MEAs that showed signs of non-random distribution. Using the specified element(s) as the center, d_{max} represents the maximum radius up to which the cluster analysis tool searches for the same element(s). N_{min} is the minimum number of ions present in the cluster.

Parameter	Ti-30Zr-14Nb-1O	Ti-30Zr-14Nb-3O	Ti-30Zr-26Nb-3O
d_{max} (nm)	0.89	0.89	0.89
N_{min}	23	23	23

Supplementary Table 2 Charge transfer (variation of the Bader charge) of metallic atoms in the Ti-30Zr-14Nb and Ti-30Zr-14Nb-O MEAs with O atoms occupying octahedral interstitials of Site 1 and Site 2. The positive and negative values of Bader charge reflect the number of electrons lost and received by the center atom, respectively.

System	Charge transfer (eV)			
	Ti	Zr	Nb	O
Ti-30Zr-14Nb (Site 1)	0.08	0.23	-	-
Ti-30Zr-14Nb-O (Site 1)	0.28	0.55		-1.36
Ti-30Zr-14Nb (Site 2)	0.41	-	-0.56	-
Ti-30Zr-14Nb-O (Site 2)	0.51	-	-0.38	-1.32

Supplementary Table 3 Theoretical increments of yield strength evaluated based on the effects of changes in grain size.

O concentration (at.%)	Yield strength increment of Ti-30Zr-14Nb-yO (MPa)	Yield strength increment of Ti-30Zr-26Nb-yO (MPa)
0	0	0
1	0.9	-1.0
2	1.9	-2.0
3	6.6	0
3.5	5.8	0.6
4	3.8	2.6

Supplementary Table 4 Actual oxygen contents in the oxygen-doped Ti-30Zr-xNb (x = 10, 14, 18, 22, 26 and 30 at.%) MEAs. The concentration (at.%) of oxygen was measured by a LECO Instruments inert gas fusion (IGF) machine with IR detection.

Alloys	Added oxygen (at.%)	Measured oxygen (at.%)
Ti-30Zr-10Nb	0	0.306
Ti-30Zr-10Nb-1O	1	1.215
Ti-30Zr-10Nb-2O	2	2.565
Ti-30Zr-10Nb-3O	3	3.333
Ti-30Zr-10Nb-3.5O	3.5	3.718
Ti-30Zr-14Nb	0	0.134
Ti-30Zr-14Nb-1O	1	1.412
Ti-30Zr-14Nb-2O	2	2.137
Ti-30Zr-14Nb-3O	3	3.355
Ti-30Zr-14Nb-3.5O	3.5	3.866
Ti-30Zr-14Nb-4O	4	4.352
Ti-30Zr-18Nb	0	0.344
Ti-30Zr-18Nb-1O	1	1.281
Ti-30Zr-18Nb-2O	2	2.378
Ti-30Zr-18Nb-3O	3	3.346
Ti-30Zr-18Nb-3.5O	3.5	3.866
Ti-30Zr-18Nb-4O	4	4.399
Ti-30Zr-22Nb	0	0.300

Ti-30Zr-22Nb-1O	1	1.314
Ti-30Zr-22Nb-2O	2	2.316
Ti-30Zr-22Nb-3O	3	3.395
Ti-30Zr-22Nb-3.5O	3.5	3.726
Ti-30Zr-22Nb-4O	4	4.254
Ti-30Zr-26Nb	0	0.226
Ti-30Zr-26Nb-1O	1	1.258
Ti-30Zr-26Nb-2O	2	2.404
Ti-30Zr-26Nb-3O	3	3.301
Ti-30Zr-26Nb-3.5O	3.5	3.712
Ti-30Zr-26Nb-4O	4	4.372
Ti-30Zr-30Nb	0	0.306
Ti-30Zr-30Nb-1O	1	1.198
Ti-30Zr-30Nb-2O	2	2.352
Ti-30Zr-30Nb-3O	3	3.302
Ti-30Zr-30Nb-3.5O	3.5	3.751

Supplementary Reference

1. Bader, R. F.W. Atoms in Molecule: A Quantum Theory (Oxford University Press, Oxford, 1990).
